# Characterization of auxin transporter *AUX*, * PIN* and * PILS* gene families in pineapple and evaluation of expression profiles during reproductive development and under abiotic stresses

**DOI:** 10.7717/peerj.11410

**Published:** 2021-06-22

**Authors:** Heming Zhao, Yan Maokai, Han Cheng, Mingliang Guo, Yanhui Liu, Lulu Wang, Shi Chao, Minqian Zhang, Linyi Lai, Yuan Qin

**Affiliations:** 1 Key Laboratory of Genetics, Breeding and Multiple Utilization of Crops, Ministry of Education; Fujian Provincial Key Laboratory of Haixia Applied Plant Systems Biology, Center for Genomics and Biotechnology, College of Agriculture, Fujian Agriculture and Forestry University, Fuzhou, Fujian Province, China; 2 State Key Laboratory of Ecological Pest Control for Fujian and Taiwan Crops, College of Plant Protection, Fujian Agriculture and Forestry University, Fuzhou, Fujian Province, China; 3 College of Life Science, Fujian Agriculture and Forestry University, Fuzhou, Fujian Province, China

**Keywords:** Pineapple (*Ananas comosus* L.), Auxin transporter, Gene family, Expression profile, Reproductive development, Abiotic stresses

## Abstract

Polar auxin transport in plant is mediated by influx and efflux transporters, which are encoded by *AUX/LAX*, *PIN* and * PILS* genes, respectively. The auxin transporter gene families have been characterized in several species from monocots and eudicots. However, a genome-wide overview of auxin transporter gene families in pineapple is not yet available. In this study, we identified a total of three*AcAUX* genes, 12 *AcPIN* genes, and seven * AcPILS* genes in the pineapple genome, which were variably located on 15 chromosomes. The exon-intron structure of these genes and properties of deduced proteins were relatively conserved within the same family. Most protein motifs were widespread in the AUX, PIN or PILS proteins, whereas a few motifs were absent in only one or two proteins. Analysis of the expression profiles of these genes elucidated that several genes exhibited either preferential or tissue-specific expression patterns in vegetative and/or reproductive tissues. * AcAUX2* was specifically expressed in the early developmental ovules, while * AcPIN1b* and * AcPILS2* were strongly expressed in stamens and ovules. * AcPIN9b*, * AcPILS1*, * AcPILS6a*, * 6b* and * 6c* were abundantly expressed in stamens. Furthermore, qRT-PCR results showed that several genes in these families were responsive to various abiotic stresses. Comparative analysis indicated that the genes with close evolutionary relationships among pineapple, rice and * Arabidopsis* exhibited similar expression patterns. Overexpression of the *AcAUX1* in * Arabidopsis* rescued the phenotype in * aux1-T*, and resulted in increased lateral roots in WT. These results will provide new insights into auxin transporter genes of pineapple and facilitate our understanding of their roles in pineapple growth and development.

## Introduction

The phytohormone auxin, one of the most essential signaling molecule, is an important regulator of a surprising variety of plant growth and development events, such as primordium differentiation, vascular tissue formation, organogenesis, gametophyte genesis, tissue regeneration, and tropisms (Friml & Palme, 2002; Swarup & Bennett, 2003; Jain et al., 2006; Petrasek & Friml, 2009; Swarup & Péret, 2012; Sun et al., 2015; Swarup & Bhosale, 2019). But not only that, auxin also plays critical role in temporal coordination of plants’ responses to many environment stresses (Wang et al., 2010; Min et al., 2014; Xie et al., 2015). The realization of the regulation function of auxin largely depends on spatiotemporal asymmetric dynamic distribution within different plant tissues through polar auxin transport system (Friml, 2003; Tanaka et al., 2006). Many previous studies have demonstrated that polar auxin transport from cell to cell is mediated through the auxin influx carriers (AUX/LAXs) and efflux carriers (PINs) (Friml et al., 2002; Blakeslee et al., 2004; Petrásek et al., 2006; Swarup et al., 2008; Habets & Offringa, 2014; Wang et al., 2015; Omelyanchuk et al., 2016).

AUX/LAXs, which encode multimembrane-spanning transmembrane proteins and locate at the upper end of plasma membrane, are required for auxin uptake and intercellular auxin flow (Marchant et al., 2002; Petrásek et al., 2006; Swarup et al., 2008; Zazímalová et al., 2010; Péret et al., 2012). PIN proteins, which are typically polar-localized on either the lower end of plasma membrane or endoplasmic reticulum (ER), enable auxin to flow out of cell. Moreover, seven ER-localized PIN-LIKES (PILS) proteins have also been found to resemble the predicted topology of PIN proteins and contribute to intracellular auxin homeostasis (Feraru et al., 2012; Sun et al., 2020). Therefore, it is generally believed that AUX/LAXs, PINs and PILSs are the main components of polar auxin transport system in plant cells, which is responsible for auxin transport from the site of biosynthesis to the sites of auxin action and the establishment of concentration gradient to achieve its regulatory function (Marchant et al., 2002; Zazímalová et al., 2010; Péret et al., 2012; Feraru et al., 2019).

The auxin influx carriers (AUX/LAX family) in higher plants belong to the auxin/amino acid permease (AAAP) family of proton-driven transporters (Saier et al., 2009; Zhao et al., 2012). The *Arabidopsis AUX/LAX* family genes encode four highly conserved transmembrane proteins named as AtAUX1, AtLAX1, 2, and 3 (Marchant et al., 2002; Swarup et al., 2004; Yang et al., 2006; Swarup et al., 2008; Péret et al., 2012). Auxin transport activity assays in heterologous expression systems confirm that all AUX/LAX proteins in *Arabidopsis* are high-affinity auxin influx transporters and mediate the uptake of the Trp-like auxin molecule indole-3-acetic acid (Bennett et al., 1996; Yang et al., 2006; Carrier et al., 2008; Swarup et al., 2008; Péret et al., 2012). *AtAUX1* and *AtLAX3* influence lateral root development by regulating primordia initiation and emergence steps, respectively (Marchant et al., 2002; Swarup et al., 2008). *At AUX1 , At LAX1* and *2* redundantly regulate the early embryo sac development, and the *aux1 lax1 lax2* triple mutant shows embryo sacs arrested at FG2 stage because of abnormal mitosis (Panoli et al., 2015). The *AUX* gene family in rice has five members, called *OsAUX1*, *2*, *3*, *4*, and *5* (Zhao et al., 2012). *OsAUX1*, a paralogous gene of *AtAUX1*, is involved in auxin influx transport and mediates lateral root initiation and response to Cd stress in rice (Zhao et al., 2015; Yu et al., 2015). Recently, *MtLAX2* has been reported as a functional analog of *AtAUX1*, and is required for the accumulation of auxin during nodule formation in tissues underlying sites of rhizobial infection (Roy et al., 2017).

The auxin efflux carrier *PIN* gene family has been first identified and characterized in detail in model plant *Arabidopsis*, which includes eight members (Paponov et al., 2005; Křeček et al., 2009). Up to date, 12 PINs in rice, 23 PINs in soybean, 12 PINs in maize, 11 PINs in sorghum and 10 PINs in potato have been identified (Shen et al., 2010; Yue et al., 2015). In *Arabidopsis*, AtPIN1-4 and AtPIN7 are located in the plasma membrane and involve in organ genesis (AtPIN1 and 4 and 7) (Friml et al., 2002; Benková et al., 2003; Reinhardt et al., 2003), gravitropism (AtPIN2 and 3) (Müller et al., 1998; Ottenschläger et al., 2003), and phototropism (AtPIN1 and 3) (Friml et al., 2002; Blakeslee et al., 2004). AtPIN5, 6 and 8 are localized in the endoplasmic reticulum, and are involved in pollen development (AtPIN5 and 8) (Dal Bosco, Dovzhenko & Palme, 2012), nectary auxin response and short stamen development (AtPIN6) (Bender et al., 2013).

In rice, *OsPIN1a* and *OsPIN1b* genes were found to be expressed in all of the tested tissues, *OsPIN9* were predominantly expressed in root and stem-base, and *OsPIN5a* and *OsPIN5b* were expressed in meristem and young panicle (Wang et al., 2009; Lu et al., 2015). In soybean, the legume-specific *GmPIN9* gene might mediate auxin redistribution in roots and be responsive to various abiotic stresses (Wang et al., 2015). In maize, the monocot-specific *ZmPIN9* was expressed in the root endodermis and pericycle, and *ZmPIN1d* transcript was observed in the L1 layer of the shoot apical meristem and inflorescence meristem during the early reproductive development (Forestan, Farinati & Varotto, 2012). *StPIN2* and *StPIN4* in potato were detected in the flower, the vascular tissue of the stolons and the tuber parenchyma cells (Roumeliotis et al., 2013). *SlSoPIN1* gene in tomato (*Solanum lycopersicum*) defined leaf and flower organ initiation patterns by maintaining epidermal auxin flux (Martinez et al., 2016).

The PILS proteins have been identified to be putative auxin carriers at the endoplasmic reticulum (ER) and control intracellular auxin accumulation (Barbez et al., 2012; Feraru et al., 2012; Mohanta, Mohanta & Bae, 2015). Although PILS proteins have a low (10%–18%) sequence identity with PINs, all of them contain the InterPro auxin carrier domain, which is the same as PINs (Habets & Offringa, 2014). Auxin transport activity assay in heterologous systems has showed that PILS2, PILS3, and PILS5 function in auxin transport and sequester auxin in the ER (Feraru et al., 2012; Béziat et al., 2017). The recent study showed that PILS6 negatively regulates root growth though limiting nuclear availability of auxin (Feraru et al., 2019), and PILS genes coordinate brassinosteroid (BR) hormone signaling with nuclear auxin input in *Arabidopsis thaliana* (Sun et al., 2020).

Pineapple is a major tropical fruit cultivated in most tropical and subtropical countries and in other regions with mild climates, ranking second among tropical fruits after banana in terms of international trade (Zhang, Liu & Ming, 2014; Ming et al., 2015). Although the auxin transporters *AUX/LAX*, *PIN* and *PILS* gene families have been extensively identified and analyzed functionally in monocots and eudicots, little is known about these genes in pineapple. Therefore, there is an urgent need for a thorough bioinformatic analysis and characterization of auxin transporters in pineapple genomes. Completion of the pineapple genome sequencing makes it possible to identify polar auxin transport genes from pineapple (Ming et al., 2015). This study will provide comprehensive analyses of polar auxin transport genes in pineapple, including the identification of *AcAUX*, *AcPIN* and *AcPILS* family members, the phylogenetic relationships, gene structures, as well as their expression profiling in different vegetable organs/tissues and ovules of several developmental stages. These results will facilitate functional validation of the pineapple *AcAUX*, *AcPIN* and *AcPILS* genes and broaden our understandings of the roles of polar auxin transport genes in plants.

## Materials and Methods

### Plant materials and sampling

The pineapple (*Ananas comosus* (L.) Merr. CV. MD-2) plants were grown in a greenhouse at Fujian Agriculture and Forestry University. Plant materials for expression pattern analysis were roots, leaves, flower buds, and six different developmental stages fruits which were S1, S2, S3, S4, S5, and S6, as described by Ming et al. (2015). The four flower organs tested in the expression profile analysis during pineapple reproductive development were calyxes, petals, stamens, and ovules during different development stages, as described by Su et al. (2017) and Ali et al. (2017). The flower organs at different developmental stages were defined based on the flower buds’ diameters in pineapple inflorescences. The calyxes at four developmental stages included calyxes during early flower developmental stage (Ca1) with buds’ width 5–7 mm, calyxes before flowering (Ca2) with buds’ width 8–9 mm, calyxes when flowering (Ca3), and calyxes after flowering (Ca4). The petals at three stages were petals during early flower developmental stage (Pe1) with buds’ width 5–8 mm, petals before flowering (Pe2) with buds’ width 8-9 mm, and petals when flowering (Pe3). The five different developmental stages in stamens were St1 with buds’ width <5 mm, St2 with buds’ width 5–8 mm, St3 with buds’ width >8 mm, St4 with just showing petals, and St5 with one mm petals visible. The seven different developmental stages in ovules were Ov1 with buds’ width <5 mm, Ov2 with buds’ width 5–8 mm, Ov3 with buds’ width eight mm, Ov4 with buds’ width >8 mm, Ov5 with just showing petals, Ov6 with one mm petals visible, and Ov7 with >2 mm petal visible to pre-flowering, as described by Su et al. (2017). These flower tissues materials were taken with three replicates and quickly frozen in liquid nitrogen, and stored at −80 °C until RNA extraction.

For stress treatments, the pineapple seedlings were carefully transferred into the solution with mannitol at 28 °C as drought stress, placed in 400 mM NaCl solution at 28 °C as salt stress, and kept in sterile water at 4 °C and 45 °C as cold stress and heat stress, respectively. All the pineapple seedlings were placed in the growth chambers with a 14:10 light: dark photoperiod at different temperatures. Roots and shoots were collected separately at 6 h, 12 h, 24 h, and 48 h after treatment, and parallel control samples were prepared at each time point. Three biological replicate samples were used for this study. The harvested samples were immediately frozen in liquid nitrogen, and stored in −80 °C environment until RNA extraction.

### Database screening and identification of AcPINs, AcAUXs and AcPILSs

Two approaches were employed for the mining of all putative *AUX*, *PIN* and *PILS* auxin transporter familiy members in the pineapple genome. Firstly, BLASTP searches of the consensus protein sequences (PF01490, Pfam 03547 and Pfam PF03547) search were performed in phytozome (http://www.phytozome.net/). Secondly, protein sequences of putative *AUX*, *PIN* and *PILS* family members were downloaded from Search Interpro (http://www.ebi.ac.uk/ interpro/ISearch?query = PF01490 and PF03547). Resulting protein sequences were then used as queries to perform two database searches against MSU-RGAP (http://rice.plantbiology.msu.edu/) and NCBI (http://www.ncbi.nlm.nih.gov/). After removing the redundant sequences, the remaining protein sequences were then used as queries to screen for the presence of the conserved membrane transport protein domain using InterProScan Sequence Search (http://www.ebi.ac.uk/interpro/search/sequence-search). The *AcAUX*, *AcPIN* and *AcPILS* genes in pineapple were named according to sequence homology to these genes in rice and *A. thaliana*. Information about genomic DNA, full-length cDNA, coding sequence length for each gene and characteristics of proteins were obtained from phytozome (http://www.phytozome.net/). The predicted intron-exon structures of *AcAUXs*, *AcPINs* and *AcPILSs* were retrieved on the website of GSDS (Gene Structure Display Server) (http://gsds.cbi.pku.edu.cn/). Prediction of the putative transmembrane regions in AcAUX, AcPIN and AcPILS proteins was performed with the TMHMM Server v2.0 (http://www.cbs.dtu.dk/services/TMHMM/).

### Chromosomal localization

The physical positions of *AcAUXs*, *AcPINs*, and *AcPILSs* genes were used to map these genes onto the corresponding pineapple chromosomes. The distribution of *AcAUXs*, *AcPINs*, and *AcPILSs* genes on chromosomes was drawn by using the MapChart software (Voorrips, 2002) and modified manually with annotation.

### Phylogenetic analysis and sequence alignment

The multiple sequence alignment was performed based on amino acid sequences of AUX, PIN and PILS proteins by using ClustalX version 1.83, and the phylogenetic tree based on the full-length protein sequences of AUX, PIN and PILS proteins in pineapple, *Arabidopsis* and rice was constructed using MEGA 6.06 with 1000 bootstrap replications by Maximum likelihood method, Jones-Taylor-Thornton (JTT) model and complete deletion (Tamura et al., 2007; Cao et al., 2019). The combined trees with AUX, PIN and PILS proteins in pineapple, *Arabidopsis*, sorghum, and Brachypodium distachyon were generated using the same method, respectively. The conserved motifs within AUX, PIN and PILS proteins in pineapple, *Arabidopsis* and rice were identified by using the MEME motif search tool (https://meme-suite.org/meme/index.html) with default settings, except that the maximum number of motifs was defined as 10 and the maximum width was set to 100. The sequence conservation of *AUX*, *PIN* and *PILS* family members in amino acid was analyzed by DNAMAN software (Ma et al., 2011) and modified manually. The conserved amino acid residues and transmembrane (TM) domains were annotated according to DNAMAN analysis and TMHMM prediction, respectively.

### Comprehensive expression analysis of *AcAUXs*, *AcPINs* and *AcPILSs*

RNA-Seq data of roots, leaves, flower buds, and five different developmental stages fruits were downloaded from NCBI (https://www.ncbi.nlm.nih.gov/Traces/study/?acc=SRP067011). Total RNA from different flower tissues in pineapple were extracted using Plant RNeasy Mini kit (Qiagen, Hilden, Germany) according to the manufacturer’s instructions. 1µg of RNA was used for cDNA synthesis and the final product was diluted 5 times in a total volume of 100 µl. The cDNA libraries for sequencing were constructed using the NEBNext Ultra™ RNA Library Prep Kit for Illumina (NEB). The transcriptome sequencing was performed on HiSeq 2500 platform using 100 bp pair-end protocols at this research center. The total reads acquired and sequencing depth in all samples were listed in Table S4. All clean reads from each sample were mapped to the reference genome with Tophat. Cufflinks was used to assemble transcripts and estimate their abundances in RNA-Seq samples (Trapnell et al., 2012). The RNA-Seq data preprocessing was performed as described by Trapnell et al. (2012) and Dai et al. (2017). The FPKM values in different tissues represented the expression values of *Ac AUX*, *AcPIN*, *AcPILS* genes. To make the signal values suitable for cluster display, all the original signal values were added by 1 to get the modified vaules, which were divided by the average of all of the values. The log modified values were used to conduct the hierarchical cluster display. For the comparative expression analysis of *AUX*, *PIN* and *PILS* genes in pineapple, *Arabidopsis* and rice, microarray expression data of these genes in *Arabidopsis* and rice were also downloaded from TAIR (http://www.arabidopsis.org/) and RiceGE (http://natural.salk.edu/database/RiceGE/) website, respectively. The hierarchical cluster displays were used to show the logarithmic values of the ratios from the previous step. According to the log values, expression levels of these genes were classified into four grades: high (more than 1), moderate (between 0 and 1), low (between −1 and 0), and extremely low (lower than −1), as described by Ma et al. (2011).

### Identification of transgenic *Arabidopsis* plants with overexpression of *AcAUX1*

For the overexpression construct, the 1224 bp fragment of *AcAUX1* coding sequence was amplified by PCR from the cDNA of pineapple root with the primer set *AcAUX1* OE-FP (5′ggccgcccccttcaccATGCTGTCGGGGGTGCTG3′) and *AcAUX1* OE-RP (5′cggcgcgcccaccctt TGGATGCTGCAGCGGG3′). The PCR products were then constructed into pENTER/ D-TOPO vector (Invitrogen) and then the positive clones were recombined with the destination vector pGWB505 by LR Clonase II enzyme (Invitrogen). The 35S:*AcAUX1* recombinant construction was transformed into wild-type and *aux1-T* (SALK_020355C, Da Costa, Offringa & Fett-Neto, 2020) *Arabidopsis* (Columbia ecotype) using the Agrobacterium- mediated floral dip method. Then, the expression level of *AcAUX1* in T1 generation positive seedlings was determined by qPCR. The T1 generation seeds were harvested individually to obtain T2 generation seedlings of different lines. The numbers of emergenced lateral roots in 7-day-old seedlings of wild-type, *aux1-T*, overexpression lines were observed, and student’s *t*-test was used to analyze the significant differences between WT/*aux1-T* and the overexpression lines.

### Quantitative RT-PCR analysis

To confirm the relative transcript levels of the selected genes, the total RNA extraction from all pineapple samples, cDNA synthesis and qRT-PCR was performed, as described by Ali et al. (2017) and Zhang et al. (2020). The program was: denaturation at 95 °C for 30 s; 40 cycles of annealing at 95 °C for 5s and extension at 60 °C for 35s; 95 °C for15s. At least three independent biological replicates were performed in each case. *AcActin* was used as an internal control gene, and the relative expression levels of the examined genes were evaluated using the comparative *C*_*T*_ method, as described by Su et al. (2017). Error bars indicate standard deviations of independent biological replicates (*n* = 3). The gene-specific primers were listed in Table S5. The melt curves of qRT-PCR analysis for 14 genes in this study are showed in Fig. S9.

## Results

### Identification of polar auxin transport genes in pineapple genome

To explore the entire polar auxin transport genes in pineapple, the conserved domains Pfam 01490, Pfam 03547 and Pfam PF03547 were used to identify *AcAUX*, *AcPIN* and *AcPILS* gene family numbers at Phytozome database (http://www.phytozome.net), respectively. Based on sequence similarity in proteins, *Arabidopsis* AUX/LAX, PIN and PILS proteins were used as queries employing BLAST algorithms to search against the Phytozome. After comprehensive search, a total of three *AcAUX* genes, 12 *AcPIN* genes and seven *AcPILS* genes in pineapple genome were found. The number of these auxin transport genes in pineapple was similar to that of Arabidopsis, rice, maize, and sorghum, but much less than that of Chinese cabbage and soybean (Chai & Subudhi, 2016). The *AcAUX*, *AcPIN* and *AcPILS* genes in pineapple were named according to sequence homology to these genes in rice and *Arabidopsis*. The genomic DNA, coding region and protein sequences of these genes were subsequently downloaded. The detailed information of *AcAUX*, *AcPIN* and *AcPILS* genes about gene names, locus ID, chromosome locations, intron numbers, mRNA length, ORF length for each gene, and characteristics of corresponding deduced polypeptides and the number of transmembrane regions are detailed in Table 1. In *AcAUXs* group, the three genes were located at three different chromosomes, *AcAUX1* in LG13, *AcAUX2* in LG03, and *AcAUX3* in LG17. The ORF lengths of the three genes were in the range from 1224 bp (*AcAUX1*) to 1410 bp (*AcAUX2*). The sizes of the AcAUX proteins varied slightly ranging from 407 amino acids (AcAUX1) to 469 amino acids (AcAUX2), the corresponding molecular weight varied from 45.7 kDa to 52.5 kDa, and the predicted isoelectric points varied from 8.9 (AcAUX3) to 9.45 (AcAUX2) (Table 1).

**Table 1:** The information of *AcAUX*, *AcPIN*, and *AcPILS* genes in pineapple.

Gene^a^	Accession^b^	LG-position^c^	No. of Introns^d^	Gene length^e^ (bp)	mRNA^f^ (bp)	ORF^g^ (bp)	Deduced polypeptide^h^			TM^i^
							Size (aa)	MW (Da)	pI	
**AUXs group**										
*AcAUX1*	Aco014099	LG13:78227-85094	5	6,868	2,602	1,224	407	45,700.03	9.25	10
*AcAUX2*	Aco022810	LG03:8616473-8623407	5	6,935	1,713	1,410	469	52,504.4	9.45	10
*AcAUX3*	Aco003213	LG17:1138263-1142781	7	4,519	1,493	1,392	463	52,439.34	8.9	10
**PINs group**										
*AcPIN1a*	Aco002898	LG06:12393626-12397352	5	3,727	2,310	1,878	625	67,537.25	9.65	8
*AcPIN1b*	Aco024262	LG13:3977710-3981298	5	3,589	2,066	1,794	597	64,144.22	8.97	8
*AcPIN2*	Aco006539	LG14:1507943-1510999	5	3,057	2,194	1,917	638	68,237.94	9.53	9
*AcPIN5a*	Aco017386	LG01:23954851-23962237	3	7,387	1,229	381	126	13,174.96	7.94	4
*AcPIN5b*	Aco026216	LG01:24157625-24162499	4	4,875	1,226	1,056	351	38,839.28	9.31	7
*AcPIN5c*	Aco004588	LG05:4211250-4222127	7	10,878	1,871	1,704	567	6,0728.53	9.02	11
*AcPIN5d*	Aco013144	LG25:96868-97295	2	428	243	243	80	8,416.3	10.27	2
*AcPIN6*	Aco011298	LG01:12633362-12636539	5	3,178	1,449	1,449	483	52,574.39	8.85	8
*AcPIN8*	Aco005710	LG11:12351296-12353941	5	2,646	1,456	972	323	35,271.54	9.33	7
*AcPIN9a*	Aco000614	LG12:642277-644708	5	2,432	1,236	1,236	411	44,052.05	7.69	10
*AcPIN9b*	Aco000616	LG12:623550-627250	4	3,701	1,553	1,251	416	4,4325.19	9.23	10
*AcPIN10*	Aco005423	LG11:10088855-10092754	5	3,900	2,492	1,926	641	67,686.46	6.66	9
**PILSs group**										
*AcPILS1*	Aco000734	LG02:17096147-17101044	9	4,898	1,815	1,293	430	46,267.91	8.85	10
*AcPILS2*	Aco012662	LG05:13287842-13289194	0	1,353	1,353	1,353	450	48,990	5.70	10
*AcPILS5*	Aco011923	LG03:13551090-13554622	10	3,533	1,955	1,668	555	61,947.56	8.28	10
*AcPILS6a*	Aco011167	LG04:13136639-13144201	10	7,563	2,011	1,251	416	45,010.88	9.17	10
*AcPILS6b*	Aco024698	LG21:4186817-4192853	11	6,037	1,842	1,296	431	48,019.06	8.91	7
*AcPILS6c*	Aco007145	LG23:1971977-1979952	9	7,976	2,088	1,254	417	45,758.67	8.68	9
*AcPILS7*	Aco009213	LG22:8529151-8533050	9	3,900	1,927	1,281	426	46,197.27	5.99	8

**Notes.**

a Systematic designation given to pineapple *AcAUX*, *AcPIN*, and *AcPILS* genes in this study.

b Assension identity number of pineapple *AcAUX*, *AcPIN*, and *AcPILS* genes assigned by phytozome database.

c The position of pineapple *AcAUX*, *AcPIN*, and *AcPILS* genes in linkage groups obtained from phytozome.

d Number of introns in *AcAUX*, *AcPIN*, and *AcPILS* gene encoding region.

e The genomic length of *AcAUX*, *AcPIN*, and *AcPILS* genes in pineapple.

f The length of mRNA from *AcAUX*, *AcPIN*, and *AcPILS* genes.

g The length of the open reading frame for *AcAUX*, *AcPIN*, and *AcPILS* genes.

h Protein characterization of AcAUX, AcPIN, and AcPILSs obtained from phytozome.

i The number of predicted transmembrane regions in AcAUX, AcPIN and AcPILS proteins from the TMHMM Server v2.0.

LG linkage group mRNA message RNA ORF open reading frame bp base pair aa amino acids MW molecular weight pI isoelectric point TM transmembrane

In *AcPINs* group, the twelve genes were located at eight different chromosomes, three genes (*AcPIN5a*, *AcPIN5b* and *AcPIN6*) in LG01, two genes each in LG11 (*AcPIN10* and *AcPIN8*) and LG12 (*AcPIN9a* and *AcPIN9b*), and one gene each in LG05 (*AcPIN5c*), LG06 (*AcPIN1a*), LG13 (*AcPIN1b*), LG14 (*AcPIN2*) and LG25 (*AcPIN5d*). The ORF lengths of the twelve genes were in the range from 243 bp (*AcPIN5d*) to 1926 bp (*AcPIN10*). The sizes of the AcPIN proteins varied evidently ranging from 80 amino acids (AcPIN5d) to 641 amino acids (AcPIN10), the protein molecular weight varied from 8.42 kDa (AcPIN5d) to 68.24 kDa (AcPIN2), and the predicted isoelectric points varied from 6.66 (AcPIN10) to 10.27 (AcPIN5d). It is noteworthy that the size of AcPIN5d proteins is the shortest, but the isoelectric point of this protein is the highest (Table 1). In *AcPILSs* group, the seven genes were located at seven different chromosomes, only one gene in a linkage group. The ORF lengths of the seven genes were in the range from 1251 bp (*AcPILS6a*) to 1668 bp (*AcPILS5*). The sizes of the AcPILS proteins varied slightly ranging from 416 amino acids (AcPILS6a) to 555 amino acids (AcPILS5), the corresponding molecular masses varied from 45.01 kDa to 61.95 kDa, and the predicted isoelectric points varied from 5.7 (AcPILS2) to 9.17 (AcPILS6a) (Table 1). These data showed that the sizes and molecular weight of the AcAUX, AcPIN and AcPILS proteins in the same subfamily were similar, and comparable with that of rice, and *Arabidopsis*. These characterizations indicated that members of the same subfamily were very conservative in structure among different species.

### Phylogenetic analysis of AUX, PIN and PILS families

**Figure 1:**
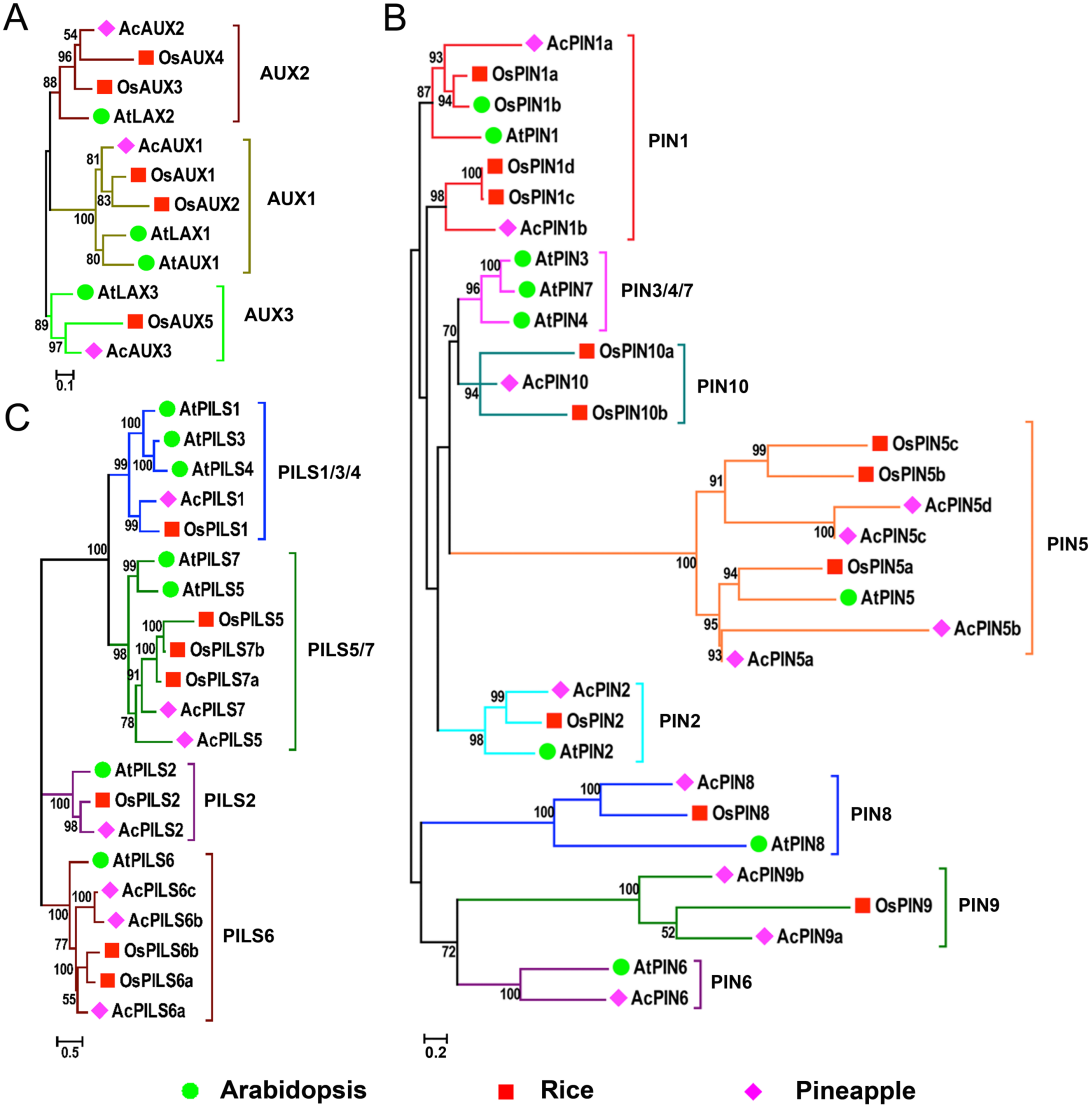
Phylogenetic relationship of AUXs, PINs and PILSs between pineapple, *Arabidopsis* and rice. Phylogenetic trees of AUX/LAXs (A), PINs (B) and PILSs (C) between pineapple, *Arabidopsis* and rice are constructed by neighbor-joining method. Bootstrap values are indicated at the nodes. The branches of different subfamilies are marked by different colors. Scale bars represent 0.1, 0.2 and 0.5 amino acid substitution per site in the trees of AUX/LAXs, PINs and PILSs, respectively. The family members from *Arabidopsis*, rice and pineapple are marked by green circle, red square and pink diamond, respectively.

In order to evaluate the evolutionary relationships and functions among *AUX*, *PIN* and *PILS* genes, three combined phylogenetic trees were generated from alignments of the full-length sequences of the AUX, PIN and PILS proteins in *Arabidopsis*, rice and pineapple using maximum likelihood method (Fig. 1). Additionally, we also constructed the phylogenetic trees of AUX, PIN, and PILS family from six species including *Arabidopsis*, rice, pineapple, maize, sorghum, and Brachypodium distachyon (Figs. S1–S3) using the same method, respectively. The 12 proteins in AUX/LAX family were classified into three distinct groups with five numbers in group AUX1, four in group AUX2, three in group AUX3, respectively (Fig. 1A). The two orthologs in rice were found in both groups AUX1 and AUX2, but only one member from pineapple was found in the three groups. Only one member form the three species was found in group AUX3, respectively.

The phylogenetic analysis on PIN family revealed that these PINs could be divided into eight distinct groups: PIN1, PIN2, PIN3/4/7 (dicot-specific), PIN5, PIN6, PIN8, PIN9 and PIN10 (monocot-specific) (Fig. 1B). Within PIN1, PIN2, PIN5 and PIN8 groups, PINs from the three species were clustered separately. More than one member from rice and pineapple were found in groups PIN1 and PIN5. The members in groups PIN9 and PIN10 were also identified from rice and pineapple. No members have been found in the PIN6 group from rice, neither do in the PIN9 and PIN10 groups from *Arabidopsis*. The monocot-specific PIN9/PIN10 groups and the dicot-specific PIN3/4/7 might have evolved independently, respectively. The change in the number of *PIN* members suggested that the *PIN* family had undergone functional divergence during the course of evolution. The PILS proteins from the three species were classified into four groups: PILS1/3/4, PILS2, PILS5/7, and PILS6 (Fig. 1C). The only one number was identified from the different species in PILS2 group, and the ortholog between AcPILS2 and OsPILS2 were more closely related than their equivalents between AcPILS2 and AtPILS2. It was clearly demonstrated that PILS3 and PILS4 in pineapple and rice were absent in PILS1/3/4 group, and the other three groups consisted of the members from these species.

### Chromosomal distribution and gene structure

**Figure 2:**
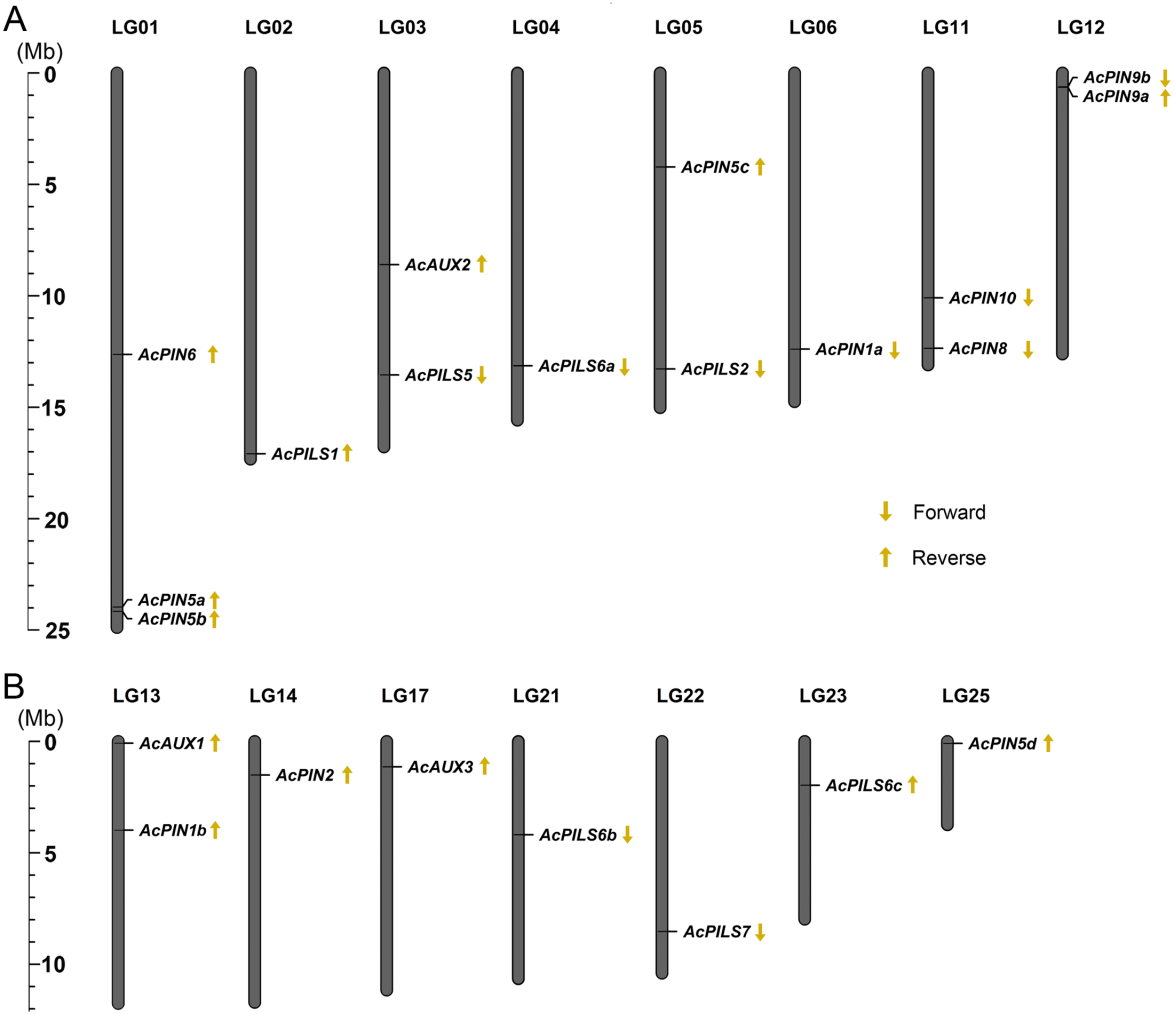
(A-B) Chromosomal localization of *AcAUXs*, *AcPINs* and *AcPILSs* genes in pineapple genome. The vertical bars represent chromosomes with the gene names shown on the right. Respective linkage group (LG) numbers are indicated at the top of each bar. The scale on the left is in megabases (Mb). The yellow arrows next to gene names show the direction of transcription.

To analyze the relationship between genetic distribution and gene duplication within the *AcAUX*, *AcPIN* and *AcPILS* gene family, the exact locations and orientation of these genes on the pineapple chromosomes were determined based on the Phytozome loci coordinate (http://www.phytozome.net). Chromosome mapping revealed that the 22 genes from *AcAUX*, *AcPIN* and *AcPILS* families were not evenly distributed on 15 chromosomes, and there was no substantial clustering of these genes in pineapple chromosomes (Fig. 2). The gene number on each chromosome varied from one to three, with only one gene each on chromosomes 2, 4, 6, 14, 17, 21, 22, 23 and 25, two genes each on chromosome 3, 5, 11, 12, and 13, three genes only on chromosome 1. Out of 22 genes, the orientations of 13 genes are reverse, and other nine genes are forward. Moreover, the orientation of three genes in chromosome 1 and two genes in chromosome 13 are all reverse (Figs. 2A and 2B).

To analyze gene structure, the full-length cDNA sequences and the corresponding genomic DNA sequences of each *AcAUX*, *AcPIN* and *AcPILS* genes were compared to determine the numbers and positions of exons and introns by using GSDS (http://gsds.cbi.pku.edu.cn/). The results showed that the number of introns in the coding sequences of these genes ranged from two to eleven, excepting *AcPILS* 2 gene without introns (Fig. S4). Three *AcPIN* genes (*AcPIN5d*, *6* and *9a*) and *AcPILS2* gene had no untranslated regions. Within a given subfamily, most members tended to share similar intron/exon structure and gene length. For example, the two members (*AcPIN1a*, and *1b*) in *AcPIN1* subfamily each contain five introns and six exons, and are all nearly 3700 bp in length. These data suggested that the gene structures of the members in the same subfamilies could be conservative.

### Transmembrane region prediction and multiple sequence alignment

Using the TMHMM Server v2.0 (http://www.cbs.dtu.dk/services/TMHMM/), prediction of the putative transmembrane regions in AcAUX, AcPIN and AcPILS proteins in pineapple was performed (Fig. S5). The data showed that the three proteins from AcAUX family had ten putative transmembrane regions (Fig. S5A). The number of predicted transmembrane regions in AcPIN proteins varied from two to 11 (Fig. S5B). According to the length of the central hydrophilic domain, as in *Arabidopsis*, the pineapple PIN genes were also divided into two groups: the long PINs and the short PINs. The long AcPIN proteins with longer central regions comprise four members (AcPIN1a, 1b, 2 and 10), and their transmembrane domains were located at the N-terminus and C-terminus regions. The other AcPIN proteins without long central region belonged to short PINs, except for AcPIN6, whose long central region was found. The number of predicted transmembrane regions in AcPILS proteins varied from seven to ten (Fig. S5C).

**Figure 3:**
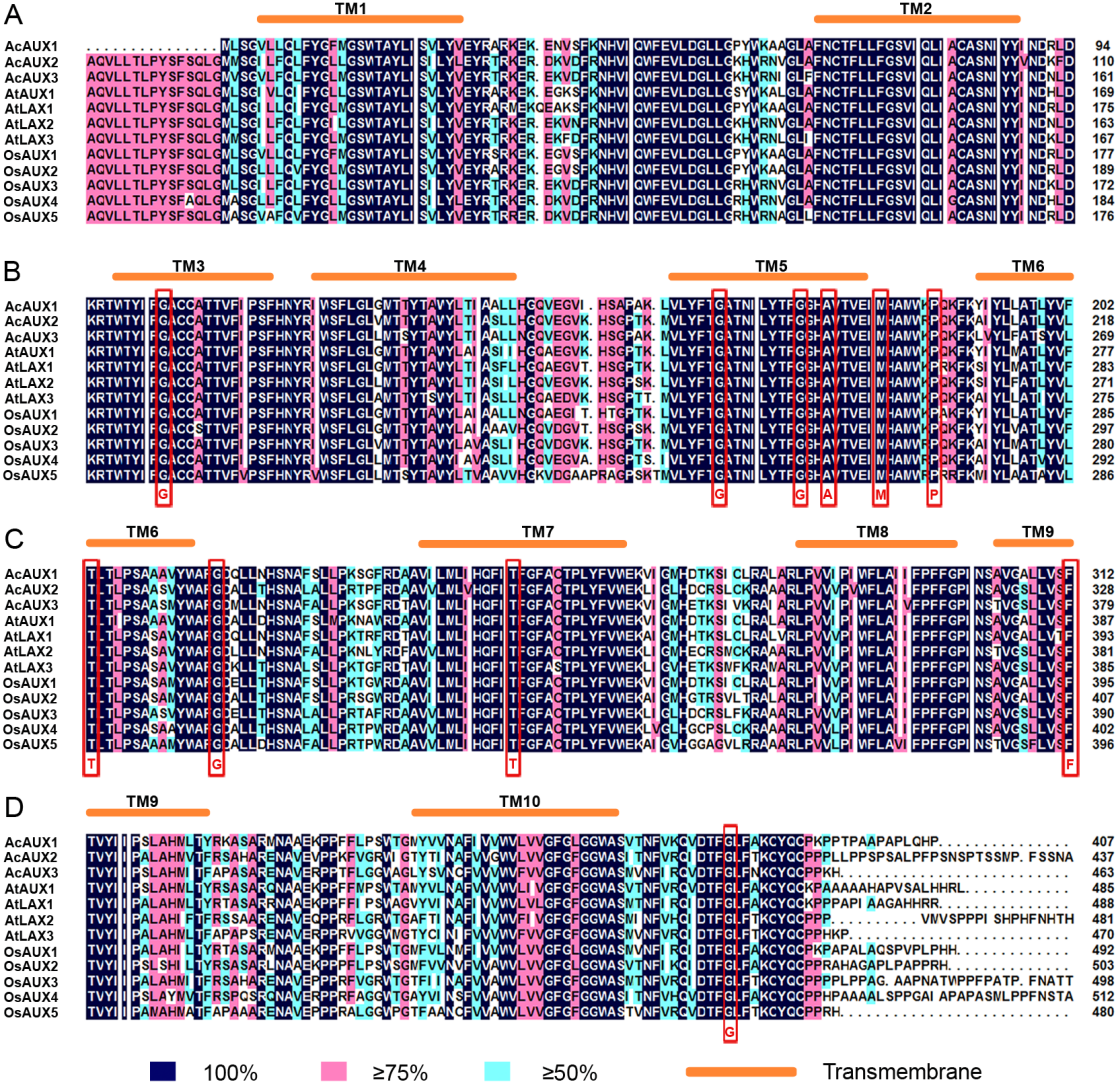
(A-D) Multiple sequence alignment of AUX/LAXproteins in **pineapple, *Arabidopsis* and rice**. Identical (100%), conservative (75–99%) and block (50–74%) of similar amino acid residues are shaded in deep blue, pink and light green, respectively. The red rectangles indicate the conserved amino acid residues known to be important for the activity of auxin influx carriers (Swarup et al., 2004). The transmembrane (TM) regions are marked by orange ellipes.

To clearly understand the sequence characteristics of AUXs, PINs and PILSs, we conducted a multiple sequence alignment using the deduced amino acid sequences of the AUX, PIN and PILS proteins from the three species (Fig. 3). The results showed that all of the AUX proteins were highly conserved. The amino acids residues that had been known to be important for the activity of auxin influx carriers (Swarup et al., 2004) were found to be completely conserved among the three *AUXs* family members (Figs. 3A–3D). The N-terminus and C-terminus regions in PIN proteins from the three species were conserved, but their central regions had no conserved sequences (Fig. S6). The alignment of the PILS proteins revealed that the conserved sequences were present in both the N- and C-terminal regions, and the central hydrophilic regions were highly variable. The major conserved domain ‘GNxGN’ at N-terminus was found, and the C-terminal regions contained two conserved domains, ‘APL’ and ‘GGNL’ (Fig. S7).

### Conserved motifs in AUX, PIN and PILS proteins

**Figure 4:**
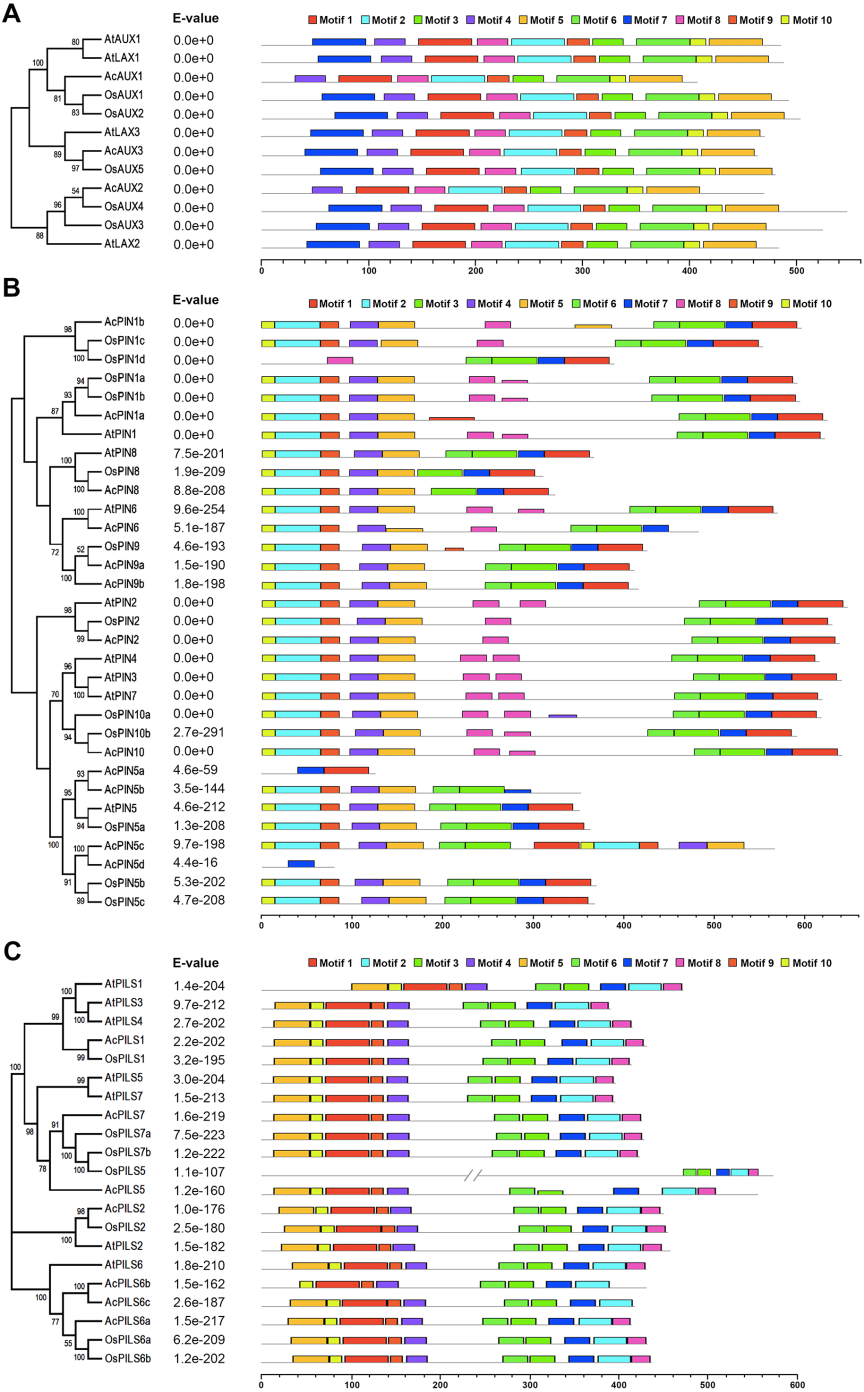
Motifs analysis of AUXs, PINs and PILSs in pineapple, *Arabidopsis* and rice. The combined phylogenetic trees of *AUX* family (A), *PIN* family (B) and *PILS* family (C) from three species are on the left panel. The motifs of corresponding proteins are shown on the right panel. Each colored box represents a specific motif in the protein identified using the MEME motif search tool. The order of the motifs corresponds to their position within individual protein sequences.

To further explore conserved motifs in AUX, PIN and PGP proteins, the MEME motif search tool was employed to identify the motifs shared in these proteins from the three species (Fig. 4, Fig. S8). As shown in Fig. 4A, the ten motifs in AUXs family were present in all of these proteins, excepting that no motif 7 were found in AcAUX1 and AcAUX2. In PINs family, the most motifs were widely distributed, and the motif 7 was completely conserved in 32 members (Fig. 4B). In contrast, the motif 8 was present in 17 of 32 proteins. The N-terminal motifs included motif 10, motif 2, motif 9, motif 4 and motif 5, and the C-terminal motifs consisted of motif 3, motif 6, motif 7 and motif 1. In AcPIN proteins, the motif 8 was absent in AcPIN1a, AcPIN5a, AcPIN5c, AcPIN5d, AcPIN8, AcPIN9a, AcPIN9b and AcPIN5b. Interestingly, AcPIN5a and AcPIN5d comprised motif 7 or/and motif 8. In PIILs family, the conserved motifs were distributed at N-terminus and C-terminus regions. AcPILS6b have no motif 5 at N-terminus and motif 8 at C-terminus, and the other motifs were present in AcPILS proteins except for AcPILS6c without motif 8 (Fig. 4C).

### Expression profile of *AcAUX*, *AcPIN*, *AcPILS* genes in several tissues and fruits

The study of gene expression is of great significance to reveal gene function. To analyze the global expression profiles of *Ac AUX*, *AcPIN*, *AcPILS* genes in pineapple, transcriptome sequencing in several tissues including root, leaf, flower and fruits during different developmental stages of MD2 (*Ananas comosus* (L.) Merr. CV. MD-2) was performed (Ming et al., 2015) and the Illumina RNA-Seq data of MD2 was selected for expression analysis of these 22 genes in three families. The expression data of *Ac AUX*, *AcPIN*, *AcPILS* genes were represented by their FPKM values in different tissues.

**Figure 5:**
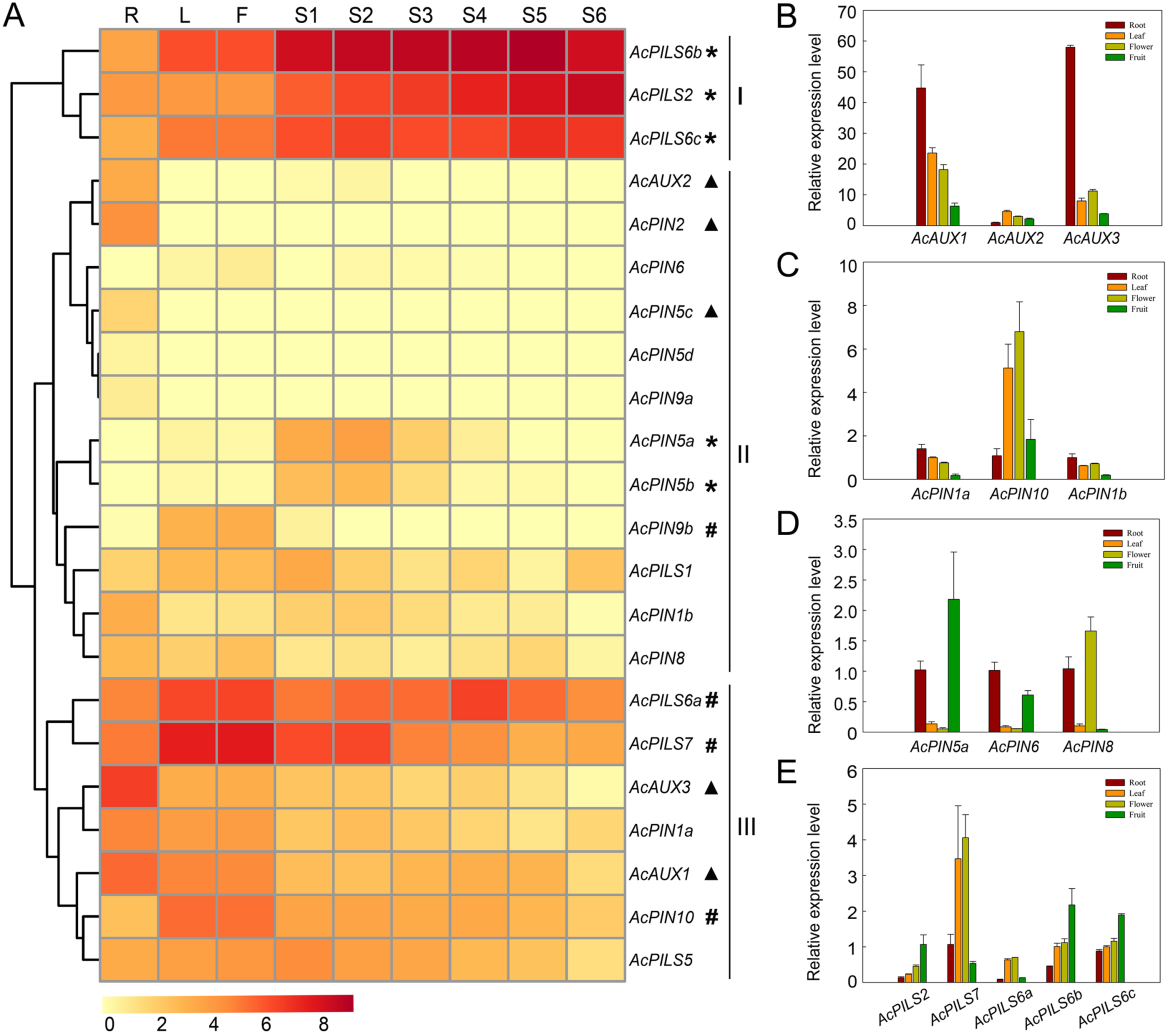
Expression analyses of *AcAUX*, *AcPIN* and *AcPILS* genes in several organs and developing fruits. (A) The heat map representing hierarchical cluster are generated by using the RNA-Seq data of these 22 genes in several organs. Color key represents average log2 expression values of the genes, red indicating high levels of transcript abundance, yellow indicating low transcript abundance , and t he colour scale is shown at the bottom. Samples are mentioned at the top of each lane: R, roots; L, leaves; F, flower; S 1 -S 5, fruit at different developmental stages. Genes that share similar expression patterns are divided into three groups: (I) preferential expression during leave, flower and fruit development; (II) low expression in the most organs; (III) high expression in specific organs . Asterisks, h ash symbols, and triangles indicate the genes with preferential expression level in fruits, leaves and flower, roots, respectively. (B)–(E) The expression pattern of *AcAUX1, 2, 3* (B), *AcPIN1a, 10, 1b* (C), *AcPIN5a, 6, 8* (D), *AcPILS2, 7, 6a, 6b, 6c* (D) in the different tissues. Error bars indicate standard deviations of independent biological replicates (*n* = 3).

The average log signal values for these 22 genes in the tissues examined were used to generate the hierarchical cluster display, and the heat map revealed the differential expression patterns of these genes, which could be divided into three major groups (Fig. 5A, Table S1). Three genes (*AcPILS6b*, *AcPILS2* and *AcPILS6c*) in group I showed relatively high expression levels in the most organs, suggesting that these genes could play important roles during pineapple growth and development. Group II comprised twelve genes that showed extremely low expression level in the most examined organs, excepting that four genes (*AcAUX2, AcPIN2, AcPIN5c* and *AcPIN1b*) were found to express moderately in roots, two genes (*AcPIN5a* and *AcPIN5b*) in S1-3 fruits, and one gene (*AcPIN9b*) in leaf and flower (Fig. 5A), suggesting that these genes might be involved in control of growth and development under specific conditions in pineapple. Group III included seven genes that showed relatively high expression level in certain organs, such as two genes (*AcAUX1* and *AcAUX3*) in roots, indicating that the genes might be involved in the regulation of root growth and development. Two genes (*AcPILS6a* and *AcPILS7*) were abundantly expressed in leaf and flower, and *AcPILS6a* in S4 fruit and *AcPILS7* in S1 and S2 fruit showed high expression level, indicating that these genes could be involved in regulating leaf, flower or fruit development in pineapple. Moreover, *AcPIN10* showed also high expression level in leaves and flowers. These results implied that those genes (*AcPILS6a*, *AcPILS6b, AcPILS6c* and *AcPILS7*) might perform function widely during the plant growth.

To confirm the expression pattern of these genes in different organs, the qRT-PCR analysis was performed in pineapple. The data showed that *AcAUX3* and *AcAUX1* were predominantly expressed in roots (Fig. 5B). The expression level of *AcPILS6a*, *AcPILS7*, and *AcPIN10* in leaf and flower were obviously higher than that in root and fruit (Figs. 5C, 5E). *AcPIN5a*, *AcPILS2*, *AcPILS6b* and *AcPILS6c* exhibited higher expression level in fruit compared to the other three organs (Figs. 5D, 5E). These results were consistent with the expression profiling in transcriptome analyses, suggesting that these genes might be involved in regulating the corresponding organs development in pineapple.

### Expression profile of *AcAUX*, *AcPIN*, *AcPILS* genes in ovules and other flower organs at different developmental stages

**Figure 6:**
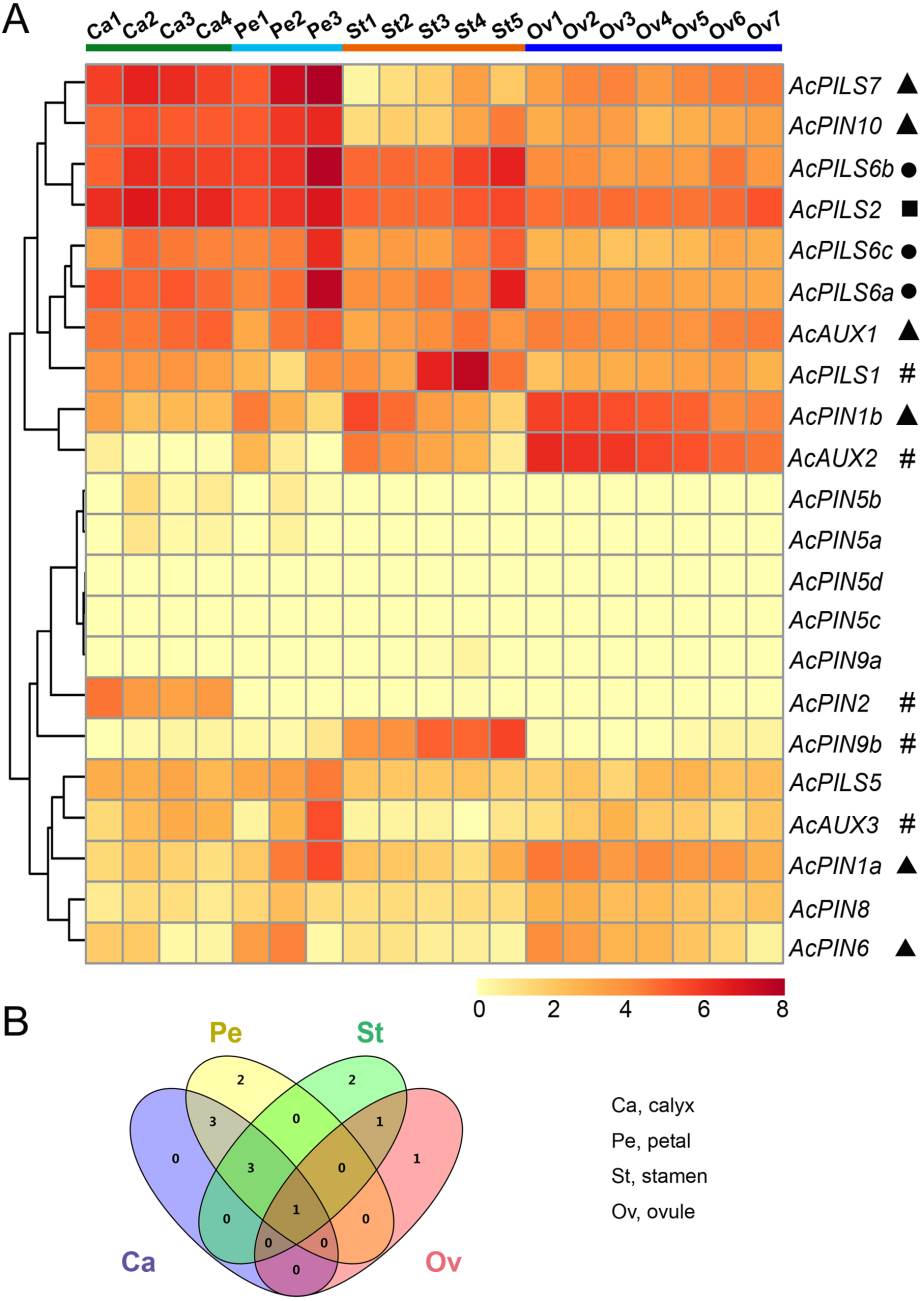
Expression profiles of *AcAUX*, *AcPIN* and *AcPILS* genes in various flower organs. (A) Expression level of *AcAUX*, * AcPIN* and *AcPILS* genes in calyxes, petals, stamens and ovules. The numbers following the tissues indicated different developmental stages. Hash symbols, triangles, rounds and squares indicate the genes with preferential expression level in only one, two, three or four types of organs, respectively. (B) Venn diagram analysis of data in (A). Summary of *AcAUX*, *AcPIN* and *AcPILS* genes expression in calyxes, petals, stamens and ovules.

To further explore putative functions of auxin transport genes in reproductive organs during different development stages in pineapple, expression patterns of *Ac AUX*, *AcPIN*, *AcPILS* genes in ovules and other flower organs were researched by using transcriptome sequencing through the Illumina HiSeq 2500 platform (Su et al., 2017). Three biological replicates for each ovule and other flower organ sample were used in this study. As described above, the FPKM values of *Ac AUX*, *AcPIN*, *AcPILS* genes in different reproductive organs including calyxes, petals, stamens and ovules were selected for hierarchical clustering analysis (Fig. 6, Table S2).

The divergent expression patterns of *Ac AUX*, *AcPIN*, *AcPILS* genes were detected in this analysis. As shown in Fig. 6A, the very high transcript levels of *AcPILS2* gene were observed in all of the four tissues (calyx, petal, stamen and ovule), while a slightly high level of *AcPILS2* was observed in stamen and ovule at their early development stages, which indicated that *AcPILS2* may be involved in all flower organ development. Similar expression patterns were observed in *AcPILS6a*, *AcPILS6b* and *AcPILS6c*, which showed relatively high expression level in calyxes, petals and mature stamens but low expression in ovules, suggesting that they could function in calyxes, petals and stamens development. *Ac AUX1* showed relatively high expression in calyxes and petals at their late development stages. Similarly, *AcPIN10* and *AcPILS7* exhibited high expression in calyxes and petals at the different development stages, but low expression in stamens and ovules. On the contrary, *AcPIN1b* had relatively high expression in petals, stamens and ovules at the early development stages. *Ac AUX2* was highly expressed in ovules at the early development stages but extremely low expression in mature ovules (Ov7), suggesting that *Ac AUX2* could play specific roles in early ovules development in pineapple. Additionally, *AcPIN2* was highly expressed only in calyxes, and *AcPIN9b* and *AcPILS1* exhibited predominantly expression level only in stamens. *AcAUX3* and *AcPIN1a* showed specially higher expression level in mature petal (Pe3) compared with the other tissues, indicating that *AcAUX3* and *AcPIN1a* might regulate petal development during late stages in pineapple (Fig. 6A). In brief, during reproductive development in pineapple, only one gene (*AcPILS2*) was abundantly expressed in four types of tissues, three genes (*AcPILS6a*, *6b*, *6c*) in three types of tissues, four genes (*AcAUX1*, *AcPIN10*, *AcPILS7* and *AcPIN1b*) in two types of tissues, and five genes (*AcPIN9b*, *AcPILS1*, *AcAUX2*, *AcAUX3* and *AcPIN1a*) in only one type of tissues (Fig. 6B). These results indicated that these auxin transport genes might be involved in the regulation of different flower organs during reproductive development in pineapple.

### Differential responses of *AcAUX*, *AcPIN* and *AcPILS* genes to abiotic stresses

**Figure 7:**
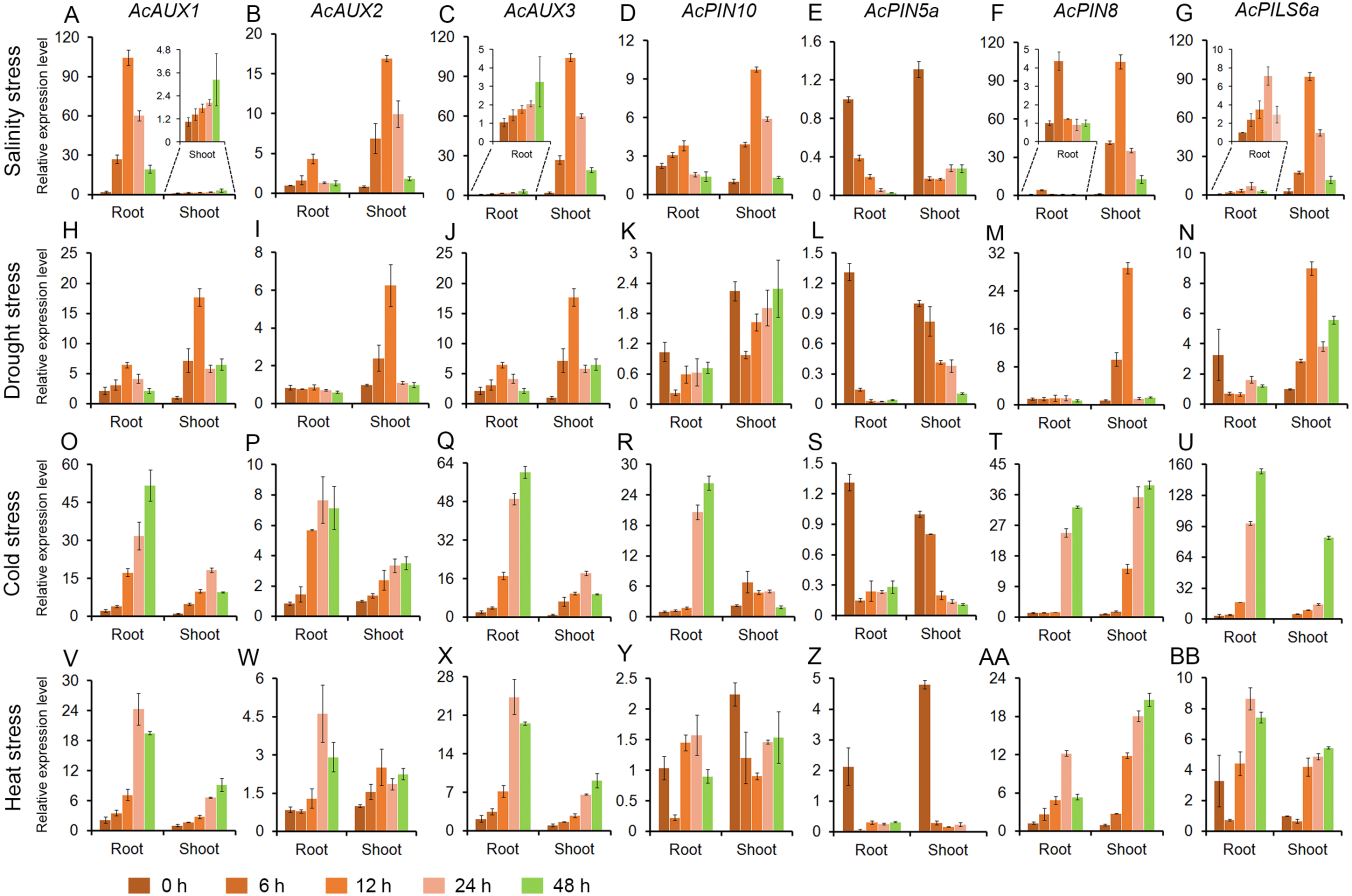
Expression profiles of the candidate genes under different abiotic stresses. These figures show the relative expression level of each selected candidate genes in different tissues (roots and shoots) and at different time point (0, 6, 12, 24 and 48 h) under salinity (A–G), drought (H–N), cold (O–U) and heat (V–BB) stress treatments. Data were normalized to *AcACTIN* gene. All qRT-PCR were performed with three biological repeats.

To address whether polar auxin transporter genes are involved in abiotic stress responses in pineapple, the expression profiles of seven genes were evaluated in roots and shoots under different abiotic stress treatments. Total RNA of all samples were isolated from the roots and shoots in pineapple seedlings treated with salt, drought, cold and heat, and the expression level of the seven genes were investigated using quantitative RT-PCR. The results showed that the expressions of the four genes (*AcAUX1*, *AcAUX2*, *AcAUX3*, and *AcPIN8*) were up-regulated both in roots and shoots under the four abiotic stresses. On the contrary, the gene *AcPIN5a* was down-regulated under the abiotic stresses, as well as *AcPIN10* and *AcPILS6a* in roots under the drought stress (Fig. 7).

Under salt stress treatment, six of the seven auxin transporter genes were evidently induced in roots and shoots, and the transcription levels of the most genes peaked at 12 h time point. And only one gene *AcPIN5a* was obviously reduced in roots and shoots (Fig. 7A). For the drought treatment, the two genes (*AcAUX1* and *AcAUX3*) were up-regulated both in roots and shoots. The transcripts of *AcAUX2* and *AcPIN8* increased significantly in shoots at the early time points, but no change in shoots at 24 h and 48 h and in roots. *AcPIN10* and *AcPIN5a* were down-regulated both in roots and shoots throughout the treatment time course, with the exception of *AcPIN10* in shoots at 48 h. *AcPILS6a* was down-regulated in roots, but up-regulated in shoots under the drought stress (Fig. 7B).

In the cold treatment, except for *AcPIN5a*, the other six genes were continuously increased in roots and shoots throughout the treatment time course (Fig. 7C). Similarly, the four genes (*AcAUX1*, *AcAUX2*, *AcAUX3*, and *AcPIN8*) were also continuously induced in roots and shoots under heat stress. Interestingly, *AcPILS6a* was down-regulated in roots and shoots at 6 h time point, but up-regulated at 12 h, 24 h and 48 h under the high temperature treatment (Fig. 7D).

Comparison of the induction kinetics of the seven genes following treatment with different abiotic stresses revealed that the expression levels of the most genes increased under the four stress treatments, such as the three auxin influx transporter genes (*AcAUX1*, *AcAUX2* and *AcAUX3*). The three genes (*AcPIN10*, *AcPIN8*, and *AcPILS6a*) showed different responses to the stresses in roots and shoots and at different time points. These data suggested that the polar auxin transporter genes had different induction kinetics in response to the abiotic stresses and could play a complex role in environmental signaling.

### Comparative expression analysis of *AUX*, *PIN*, *PILS* genes in pineapple, rice and *Arabidopsis*

To investigate valuable clues for the study of gene function, a comparative analysis of the expression patterns of *AUX*, *PIN* and *PILS* genes in pineapple, rice and *Arabidopsis* was performed by using the average log signal values from RNA-Seq and microarray data of several tissues, including roots (R), leaves (L), flowers (F), and fruits or seeds/siliques (S).

**Figure 8:**
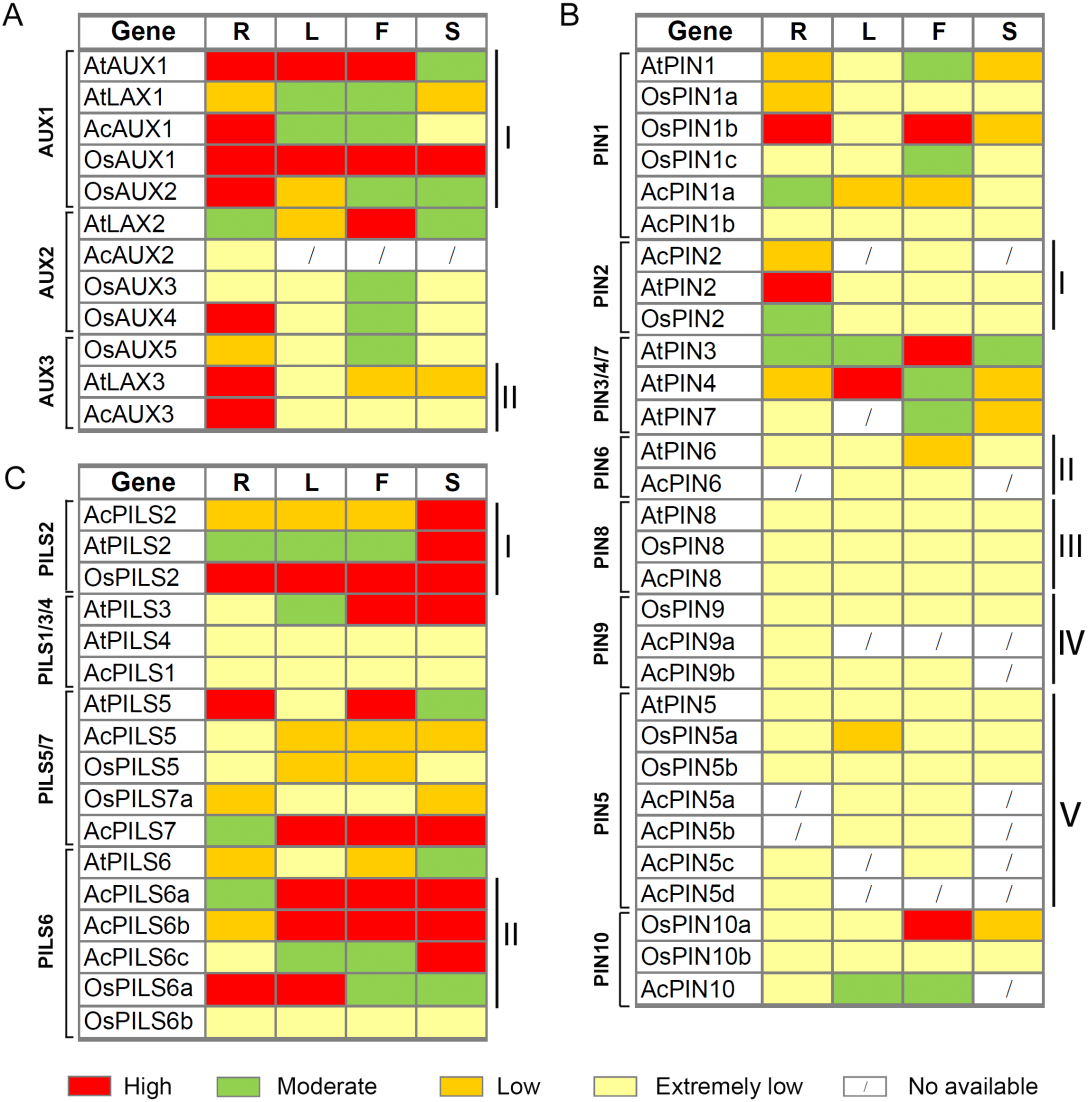
Expression comparisons of *AUX*, *PIN* and * PILS* genes between pineapple, *Arabidopsis* and rice in different organs. The *AUX* (A), *PIN* (B) and *PILS* (C) genes are displayed according to the order in the corresponding phylogenetic tree s (Fig. 1). The expression data of rice and *Arabidopsis* genes in different organ s are o b ta ined from microarray data in Rice Genome Express Database (http://signal.salk.edu/cgi-bin/RiceGE) and TAIR (http://www.arabidopsis.org/) website, respectively. The ratios of the absolute values divided by the average of all expression values were used for analysis (Table S4). Red, green, yellow and light yellow boxes indi cat e high (more than 1), moderate (between 0 and 1), low (between −1 and 0), and extremely low (less than −1 or no signature is found) expression level s, respectively. R, root; L, leaf; F, flower; S, silique, seed or fruit.

After integrating the expression data from pineapple, rice and *Arabidopsis*, we found that several genes with close evolutionary relationships showed similar expression patterns in the three species (Fig. 8, Table S3). For example, four genes (*AtAUX1*, *AcAUX1*, *OsAUX1* and *OsAUX2*) in *AUX1* group were highly expressed in roots and were moderately or high expressed in leaves and flowers (Fig. 8AI). Two genes (*AtLAX3* and *AcAUX3*) in *AUX3* group were highly expressed in roots and were low or extremely low expressed in the other organs (Fig. 8AII). In *PIN2* group, the expression levels of three genes were low or extremely low in most organs, except *AtPIN2* and *OsPIN2* that were highly or moderately expressed in roots, respectively (Fig. 8BI). All genes in *PIN6*, *PIN5*, *PIN8* and *PIN9* groups exhibited low or extremely low expression levels in all tissues examined (Fig. 8BII–Fig. 8BV). Three genes in *PILS2* group were highly expressed in fruits or seeds/ siliques, and *OsPILS2* showed high expression level in all other tissues (Fig. 8CI). In some cases, the expression pattern of several genes in pineapple differed from their *Arabidopsis* and rice homologs. For example, in *PILS6* group, the expression levels of three *AcPILS* genes from pineapple were moderately or highly in most organs (Fig. 8CII), while *AtPILS6* from *Arabidopsis* and *OsPILS6b* from rice were expressed at low or extremely low levels in the organs. These data revealed that functions of auxin transport genes in pineapple, rice and *Arabidopsis* could be conserved and divergent in the due course of growth and development. Our comparative analysis of the expression patterns of these genes will provide a foundation for future functional studies of auxin transport genes in pineapple, rice and *Arabidopsis*.

### Overexpression of *AcAUX1***induces the formation of lateral roots in***Arabidopsis*

**Figure 9:**
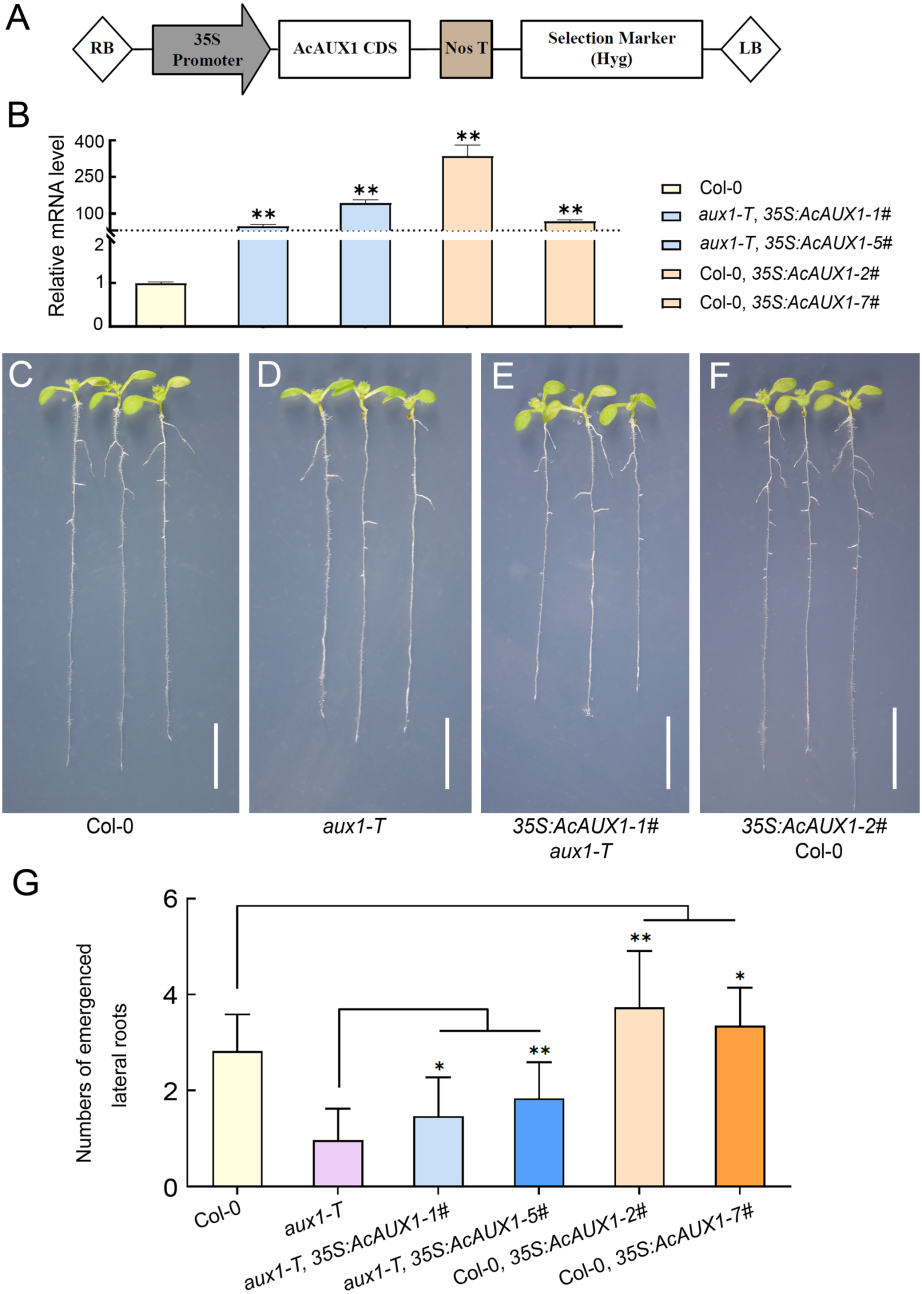
Overexpression of *AcAUX1* increased lateral roots in transgenic *Arabidopsis*. (A) Maps of the T-DNA portion of the binary vector pGWB505 used in *AcAUX1* -overexpressing construct. The *AcAUX1* coding domain sequence (CDS) used to construct the overexpression vector is indicated. RB, right border; LB, left border. Nos T, Nos terminator; Hyg, Hygromycin. (B) qRT-PCR analysis of *AcAUX1* transcripts in the transgenic *Arabidopsis* lines. *At HK2* transcripts were detected as controls. (C–F) Lateral root phenotype of 7-day-old wild-type (Col-0, C), *aux1-T* (D), overexpression lines in * aux1-T* (E) and Col-0 (F) seedlings. Bar, one cm. (G) Comparison of lateral roots in primary roots of transgenic *Arabidopsis* lines (E and F) with Col-0 (C) and *aux1-T* (D). Error bars indicate standard deviations of independent biological replicates (*n* = 20). One asterisk (*, *P* < 0.05) and two asterisks (**, *P* < 0.01) represent significant differences between the WT and transgenic lines as determined by Student’s *t* test.

As the phylogenetic analysis and multiple sequence alignment showed that the *AUX* gene family were highly conserved (Figs. 1 and 3), we asked if the function of *AcAUX1* might be similar to *AtAUX1*. To address this question, we generated transgenic *Arabidopsis* plants that overexpressed the *AcAUX1* coding region driven by the 35S promoter in Col-0 and *aux1-T* (Figs. 9A–9F). Expression analysis of the transgenic plants identified five and seven lines that overexpressed *AcAUX1* in *aux1-T* and Col-0, respectively (Fig. 9B). Overexpression of *AcAUX1* in the *aux1-T* mutant significantly increased lateral roots in the primary roots of the 7-day-old seedlings compared with *aux1-T*. Similarly, the transgenic Col-0 seedlings overexpressing *AcAUX1* formed more lateral roots than the WT (Figs. 9C–9G). Therefore, these results strongly indicated that *AcAUX1* facilitated lateral root formation in *Arabidopsis* and *AUX1* acts in lateral root development to be conserved between *Arabidopsis* and pineapple.

## Disccusion

### Characterization of *AUX, PIN* and *PILS* family genes in pineapple

Polar auxin transporters are known to play an important role in many aspects of plant growth and development. A growing body of evidence has demonstrate that the auxin influx tansporter AUX/LAXs and efflux transporter PINs coordinate to regulate polar auxin transport from cell to cell, and PILSs, which are similar to PINs protein structure, are located at the endoplasmic reticulum and contribute to maintain the intracellular auxin homeostasis (Swarup & Bhosale, 2019; Sun et al., 2020). In previous publications, several genes from *AUX*, *PIN* and *PILS* families in many species were identified and functionally characterized in detail, such as genes in the *AtAUX1/LAX* family (Swarup et al., 2008; Péret et al., 2012; Swarup & Bhosale, 2019), the *AtPIN* family (Habets & Offringa, 2014; Wang et al., 2015; Omelyanchuk et al., 2016) and the *AtPILS* family (Barbez et al., 2012; Mohanta, Mohanta & Bae, 2015; Sun et al., 2020). Considering the potential functional significance of *AUX*, *PIN* and *PILS* family members and the fact that few family members have been described in tropical crops, it was timely and quite relevant for us to characterize the *AUX*, *PIN* and *PILS* gene family in pineapple.

In this study, we identified three *AcAUX*, 12 *AcPIN* and seven *AcPILS* genes in pineapple genome. The phylogenetic analysis indicated that the numbers in the several groups from pineapple *AUX*, *PIN* and *PILS* families were similar with that in Arabidopsis and rice, and there was only one number from the three species in several groups, such as AUX3, PIN2, PIN8, PILS2 groups (Fig. 1). But, the gene numbers from pineapple in PIN5 and PILS6 groups were more than that from Arabidopsis and rice, and the largest number found in *AcPIN5* group was four. The increase in the number of AcPIN5 and AcPILS6 members reflects that expansion and rearrangement of the genome may be successfully undergone in the families, and these transporters increased in pineapple might play an important role in order to adapt to specific functions.

Chromosomal mapping of *AcAUX*, *AcPIN* and *AcPILS* family genes show their variable distribution on 15 pineapple chromosomes (Fig. 2). Gene structure analysis reveals that most members in the same subfamily are structurally conserved in the number of intron/exon and gene length (Fig. S4). The deduced amino acid sequences and motifs of the AUX, PIN and PILS proteins from the three species were highly conserved (Figs. 3 and 4), which indicates their close evolutionary relationship and the introduced classification of subfamilies.

### Expression patterns divergence and putative function of *AcAUX*, *AcPIN* and *AcPILS* Genes in vegetative growth

The analysis on spatial and temporal expression patterns of *AcAUX*, *AcPIN* and *AcPILS* genes may provide useful information for establishing their putative functions. Our transcriptome and qRT-PCR analysis showed that the expression patterns of the 22 genes could be divided into three major groups. Some preferential or tissue-specific expression *AcAUX*, *AcPIN* and *AcPILS* genes were also identified. The four (*AcAUX1*, *AcAUX2*, *AcAUX3* and *AcPIN2*) of the 22 genes were found to exhibit either preferential or tissue-specific expression in root, two (*AcPILS6a* and *AcPILS7*) in leaf and flower, another three (*AcPILS2*, *AcPILS6b* and *AcPILS6c*) in fruit (Fig. 5).

The previous reports have revealed that *AtAUX1*, *AtLAX3*, and *OsAUX1* are primarily expressed in root and promoted lateral root formation in *Arabidopsis* and rice (Zhao et al., 2015), and *AtLAX2* is also found to show the involvement in leaf venation patterning and normal xylem development (Moreno-Piovano et al., 2017). Our data showed that *AcAUX1* and *AcAUX3*, orthologs of *AtAUX1* and *AtLAX3*, were highly and specifically expressed in root, and *AcPIN2* had almost preferential expression levels in root. Overexpression of *AcAUX1* in the *aux1-T* mutant and Col-0 significantly increased lateral roots in the primary roots of the 7-day-old seedlings (Fig. 9), indicating that the function of *AcAUX1* and *AtAUX1* in lateral root development is conserved. In addition, *AcPIN10* were predominantly expressed in leaf and flower (Fig. 5), similar with *AtPIN1* (Benková et al., 2003; Reinhardt et al., 2003) and *OsPIN1* (Xu et al., 2005; Wang et al., 2009). Similar expression patterns suggest that these genes might play important roles in root, leaf and/or flower development (Figs. 5 and 8). Moreover, the multiple sequence alignment showed that the important amino acids residues for the activity of auxin influx carriers were completely conserved among the three *AUXs* family members (Fig. 3). Therefore, combining their phylogenetic relationship, we infer that *AcAUX1* and *AcAUX3* might encode putative auxin influx carriers and participate in the root formation and development, and *AcPIN10* might function in leaf and flower development in pineapple.

### Putative roles of *AcAUXs*, *AcPINs* and *AcPILSs* in reproductive development and in response to abiotic stresses

Many evidences demonstrate that auxin signaling genes, including auxin influx and efflux carriers, are associated with stress response and reproductive development, and some *AUX*, *PIN* or *PILS* genes may be co-regulated by both environmental factors and developmental cues (Cooper et al., 2003; Jain et al., 2007; Habets & Offringa, 2014; Zhao et al., 2015; Swarup & Bhosale, 2019). The expressional analysis from RNA-Seq and qRT-PCR data in this study also indicated that several genes (such as *AcAUX2*, *AcPIN10*, and *AcPILS6a*), which were differentially expressed during at least one of the flower organ developmental stages, were obviously down- or up-regulated by one or more of the stress conditions (Figs. 6 and 7).

The multiple mutants’ analysis of AUX1/LAX family members revealed that *aux1lax1lax2* triple mutant and *aux1lax1lax2lax3* quadruple mutant had multiple gametophyte defects with about 29% ovules showing aberrant embryo sacs in *Arabidopsis* (Panoli et al., 2015). It has been demonstrated that the florescence branching and spikelets in the central spike reduce in a loss-of-function allele of *ZmAUX1* termed *Zmaux1-0* in maize (Huang et al., 2017). *ZmPIN1*, an auxin efflux carrier in maize, has been also implicated to involve in florescence development (Skirpan et al., 2009). The research in rice find that *OsAUX1* and *OsAUX2*, two members in rice *AUX* family, are predominantly expressed in late florescence, and on the contrary, *OsAUX3* and *OsAUX5* are preferentially expressed in young florescence (Zhao et al., 2012). The analyses of *OsAUX1* transcripts in 7-day-old WT seedlings treated with various abiotic stresses reveal that *OsAUX1* is significantly down-regulated by drought and salt stresses (Zhao et al., 2015). Similarly, in our investigation, *AcAUX2* exhibited a preferential expression level at several early developmental stages in ovule (Fig. 6), and the qRT-PCR analysis found that this gene was up-regulated in root and shoot under the four abiotic stresses (Fig. 7). Moreover, *AcPILS6a* exhibited a preferential expression level at several developmental stages in calyx, petal and stamen, and was up-regulated in root and shoot under the abiotic stresses, except for down-regulation in root under drought stress (Figs. 6 and 7). These data suggest that the auxin transporter genes may play important roles in plant growth and response to different abiotic stress conditions during reproductive development, and a number of *AcAUX*, *AcPIN* and *AcPILS* genes might be involved in main developmental processes and stress responses. And their direct relationship requires further experimental validation.

## Conclusions

In conclusion, the results of this study display the genomic framework, characterization, phylogenetic relationship and conserved amino acid sequences of the 22 polar auxin transporter genes in pineapple. Moreover, the comprehensive expression profiles analyses revealed that several auxin transporter genes (such as *AcAUX2*, *AcPIN10* and *AcPILS6a*) might play crucial roles during reproductive development and under abiotic stresses. Overexpression of *AcAUX1* facilitated lateral root formation in *Arabidopsis*. These data could provide a solid foundation for further understanding of the functions of auxin transporter genes in pineapple growth and development, which would contribute to selecting candidate genes for functional validation studies of auxin transporter genes in pineapple.

##  Supplemental Information

10.7717/peerj.11410/supp-1 Supplemental Information 1 The phylogenetic trees of AUX family among *Arabidopsis*, rice, pineapple, maize, sorghum, and Brachypodium distachyon

10.7717/peerj.11410/supp-2 Supplemental Information 2 The phylogenetic trees of PIN family among *Arabidopsis*, rice, pineapple, maize, sorghum, and Brachypodium distachyon

10.7717/peerj.11410/supp-3 Supplemental Information 3 The phylogenetic trees of PILS family among *Arabidopsis*, rice, pineapple, maize, sorghum, and Brachypodium distachyon

10.7717/peerj.11410/supp-4 Supplemental Information 4 Intron-exon structures of *AcAUX*, *AcPIN* and *AcPILS* genes The phylogenetic trees of *Ac AUX* family (A), *AcPIN* family (B) and *AcPILS* family (C) on the left panel are constructed by maximum likelihood method. The gene structures on the right panel were drawn by using the Gene Structure Display Server (http://gsds.cbi.pku.edu.cn/). The blue boxes, UTR (Un-translated regions), orange boxes, exons; black lines, introns. The numbers on the right indicate the genomic length of the corresponding genes. bp, base pair.

10.7717/peerj.11410/supp-5 Supplemental Information 5 Prediction of transmembrane regions of AcAUX, AcPIN and AcPILS proteins The transmembrane regions of AcAUX (A), AcPIN (B) and AcPILS (C)** proteins are predicted by using the TMHMM Server v2.0 (http://www.cbs.dtu.dk/services/TMHMM/) and displayed according to the order in Table 1. The predicted transmembrane helices are shown as red peaks and blocks, the areas of the proteins predicted to be outside the cell are represented by the pink lines, and the areas of the proteins predicted to be inside the cell are represented by the blue lines.

10.7717/peerj.11410/supp-6 Supplemental Information 6 Multiple sequence alignment of PIN proteins in pineapple, Arabidopsis and rice Identical, conservative and block of similar amino acid residues are shaded in deep blue, pink and light green, respectively. The transmembrane (TM) regions are marked by orange ellipses. Asterisks () indicate the completely conserved amino acid.

10.7717/peerj.11410/supp-7 Supplemental Information 7 Multiple sequence alignment of PILS proteins in pineapple, Arabidopsis and rice Identical, conservative and block of similar amino acid residues are shaded in deep blue, pink and light green, respectively. The red rectangles indicate the conserved domain at N-terminus or C-terminus. The transmembrane (TM) regions are marked by orange ellipses.

10.7717/peerj.11410/supp-8 Supplemental Information 8 Sequences of conserved motif in AcAUX, AcPIN and AcPILS proteins The motif sequences of AUX (A), PIN (B) and PILS(C) proteins in Fig. 6 are shown. Conversed protein motifs were identified by MEME motif search tool. The height of each character in the motif sequences represent the conservation of amino acid identity.

10.7717/peerj.11410/supp-9 Supplemental Information 9 The melt curves of qRT-PCR analysis for 14 genes in this study

10.7717/peerj.11410/supp-10 Supplemental Information 10 Expression data of *AcAUX*, *AcPIN* and *AcPILS* genes in several tissues and fruits

10.7717/peerj.11410/supp-11 Supplemental Information 11 Expression data of *AcAUX*, *AcPIN* and *AcPILS* genes in various flower organs

10.7717/peerj.11410/supp-12 Supplemental Information 12 Data for expression comparison of *AUX* (A), *PIN* (B) and *PILS* (C) genes between pineapple, Arabido*psis* and rice in different organs

10.7717/peerj.11410/supp-13 Supplemental Information 13 Total reads and sequencing depth for 28 samples

10.7717/peerj.11410/supp-14 Supplemental Information 14 The gene-specific primers for qRT-PCR in this study

## Additional Information and Declarations

### Data Availability

The following information was supplied regarding data availability: The raw measurements are available in the Supplemental Files.
