# Supplementary figures and images for: Characterization of auxin transporter AUX, PIN and PILS gene families in pineapple and evaluation of expression profiles during reproductive development and under abiotic stresses
Source: PeerJ. 2021 Jun 22;9:e11410. doi: 10.7717/peerj.11410 (PMC8231336; doi:10.7717/peerj.11410)

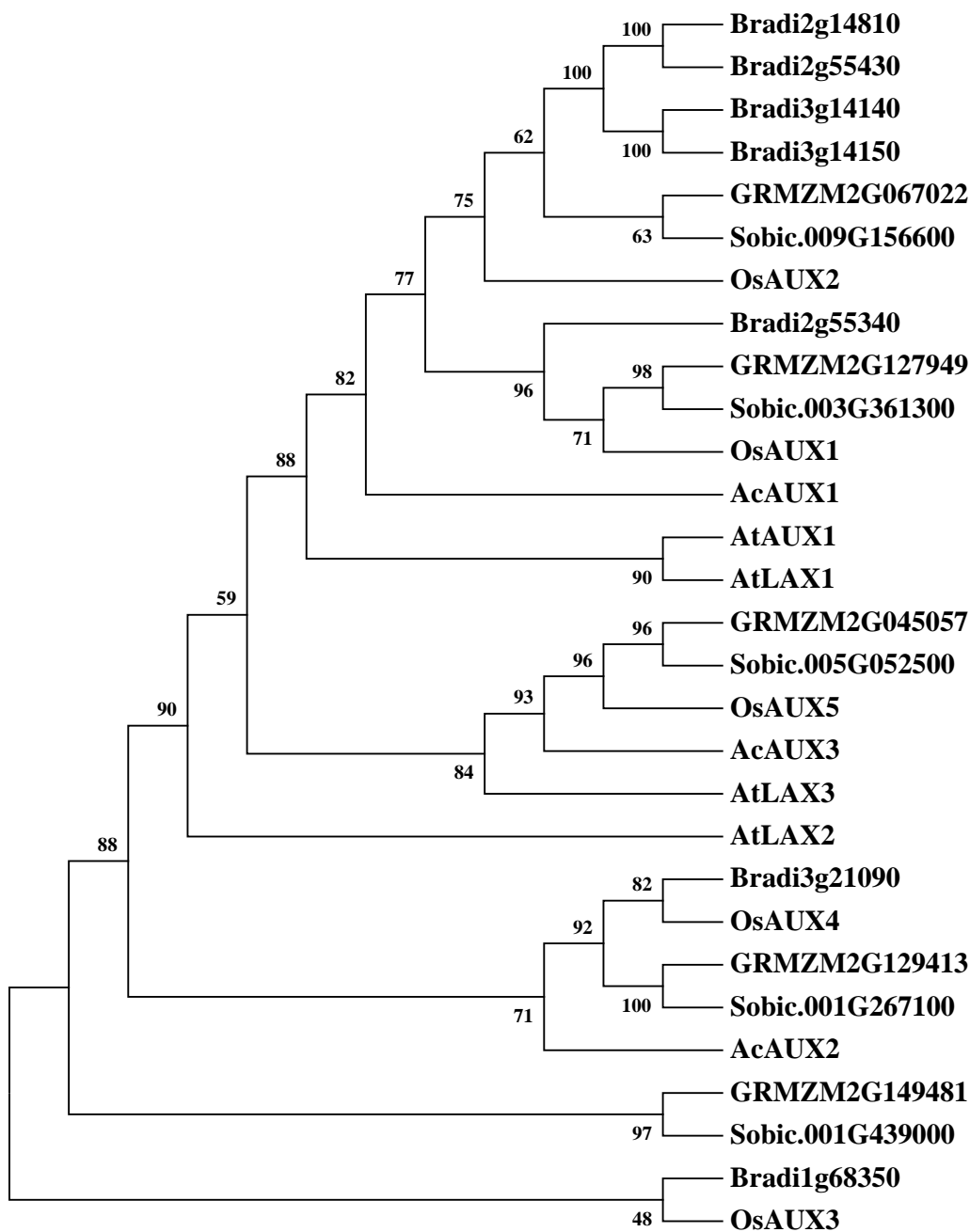

Supplement: Supplemental Information 1 [file peerj-09-11410-s001.pdf]

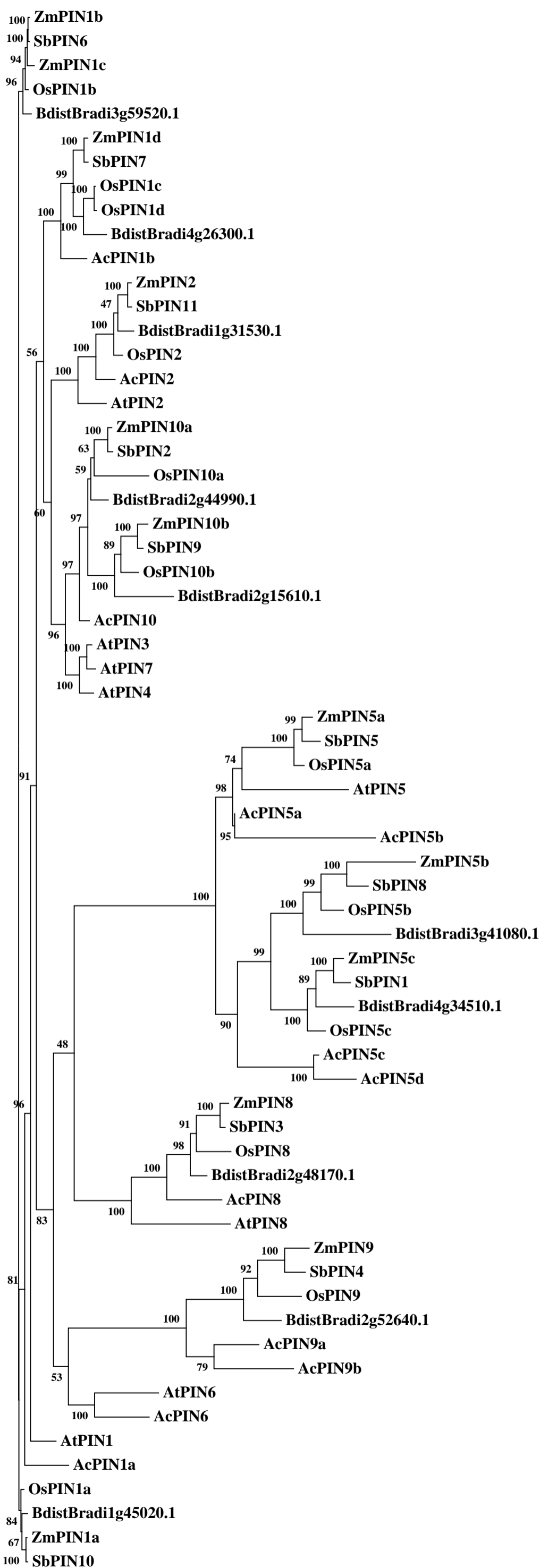

Supplement: Supplemental Information 2 [file peerj-09-11410-s002.pdf]

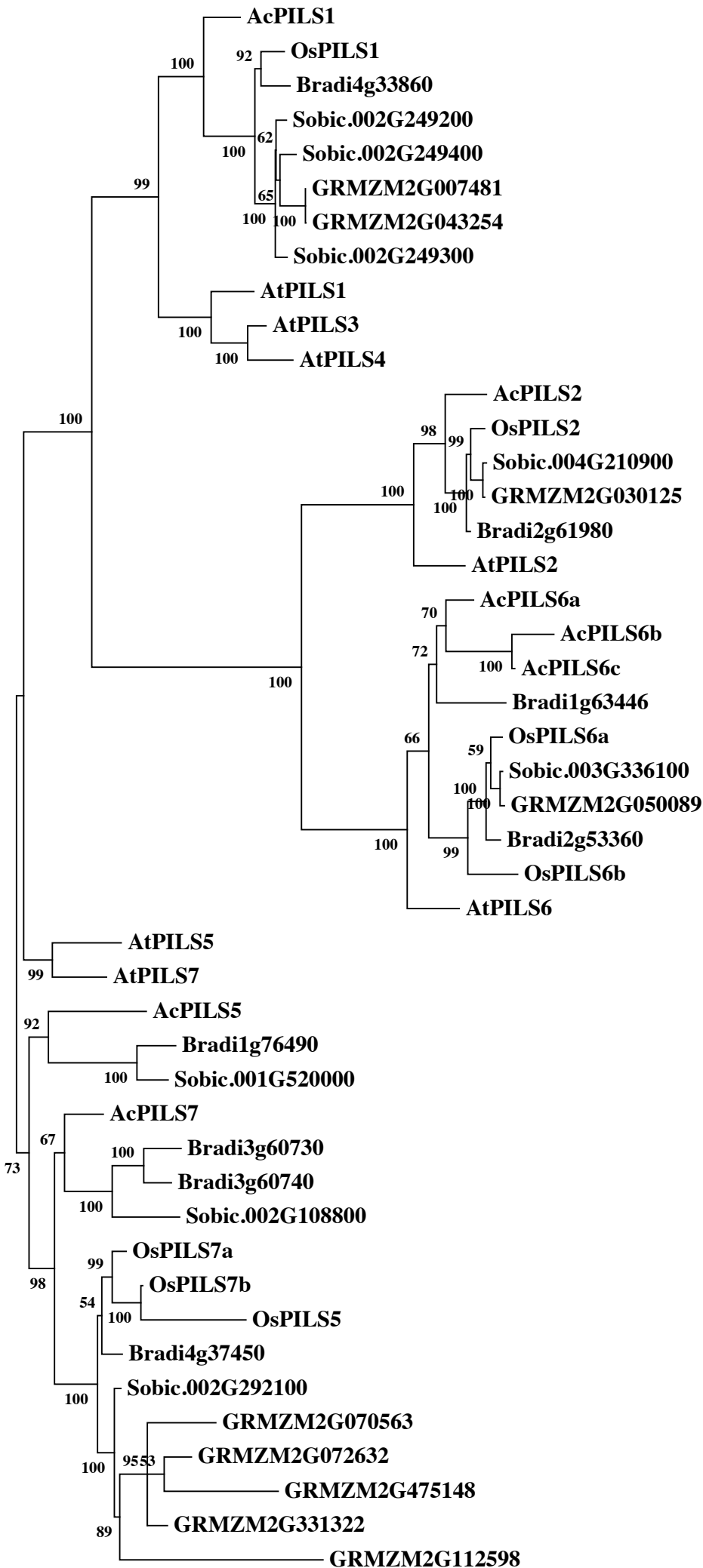

0.50

Supplement: Supplemental Information 3 [file peerj-09-11410-s003.pdf]

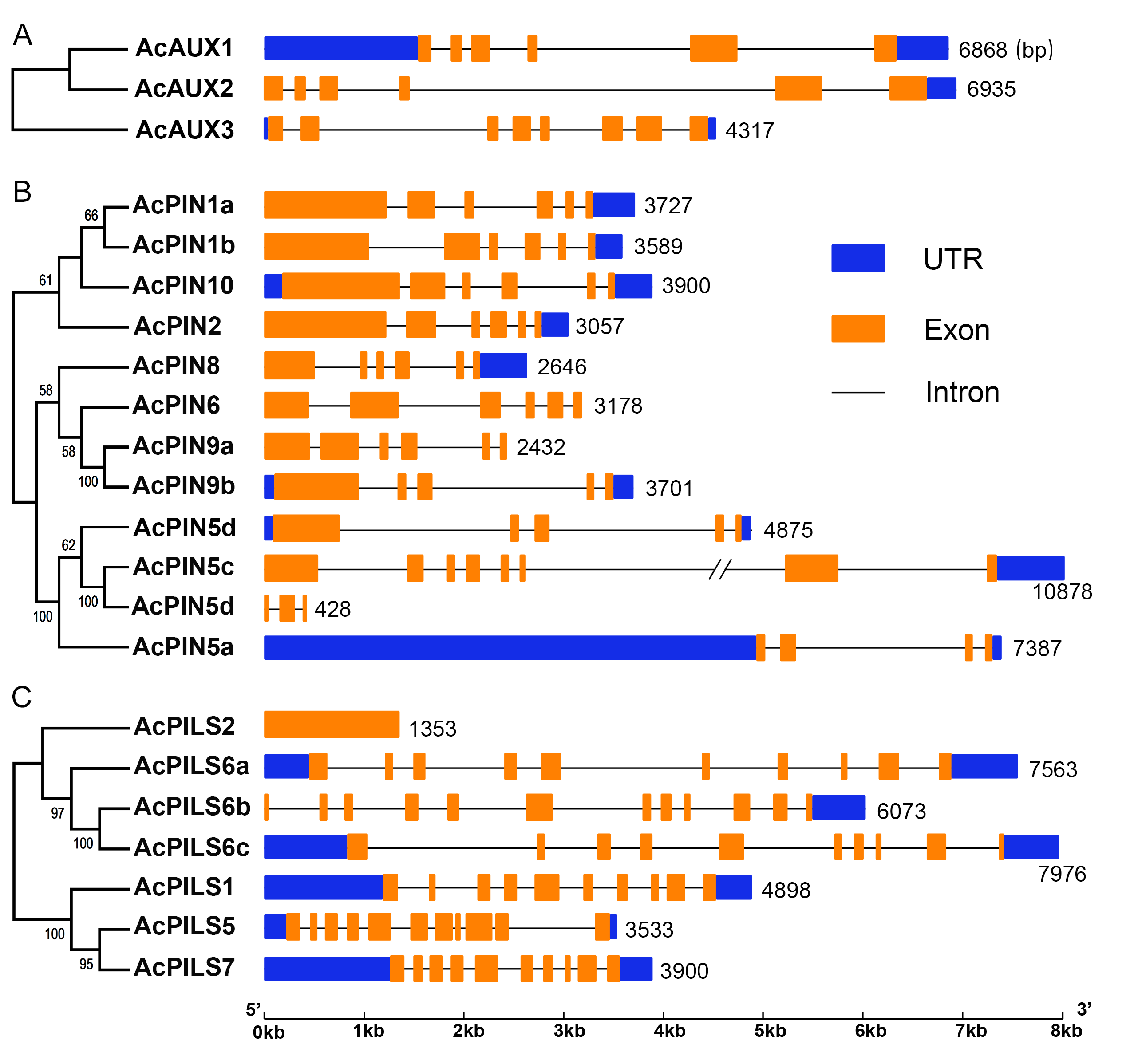

Supplement: Supplemental Information 4 — The phylogenetic trees of Ac AUX family (A), AcPIN family (B) and AcPILS family (C) on the left panel are constructed by maximum likelihood method. The gene structures on the right panel were drawn by using the Gene Structure Display Server (http://gsds.cbi.pku.edu.cn/). The blue boxes, UTR (Un-translated regions), orange boxes, exons; black lines, introns. The numbers on the right indicate the genomic length of the corresponding genes. bp, base pair. [file peerj-09-11410-s004.png]

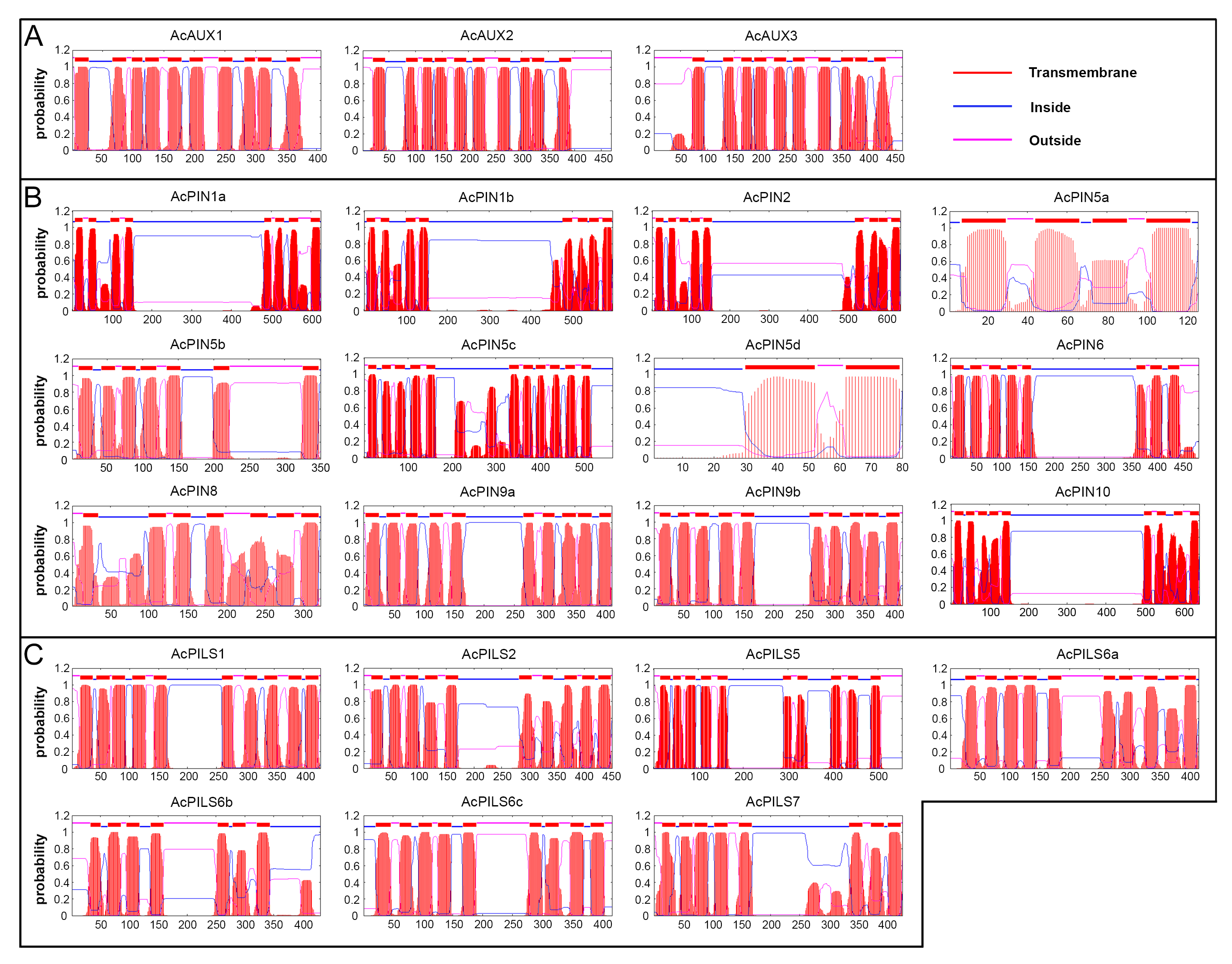

Supplement: Supplemental Information 5 — The transmembrane regions of AcAUX (A), AcPIN (B) and AcPILS (C) proteins are predicted by using the TMHMM Server v2.0 (http://www.cbs.dtu.dk/services/TMHMM/) and displayed according to the order in Table 1. The predicted transmembrane helices are shown as red peaks and blocks, the areas of the proteins predicted to be outside the cell are represented by the pink lines, and the areas of the proteins predicted to be inside the cell are represented by the blue lines. [file peerj-09-11410-s005.png]

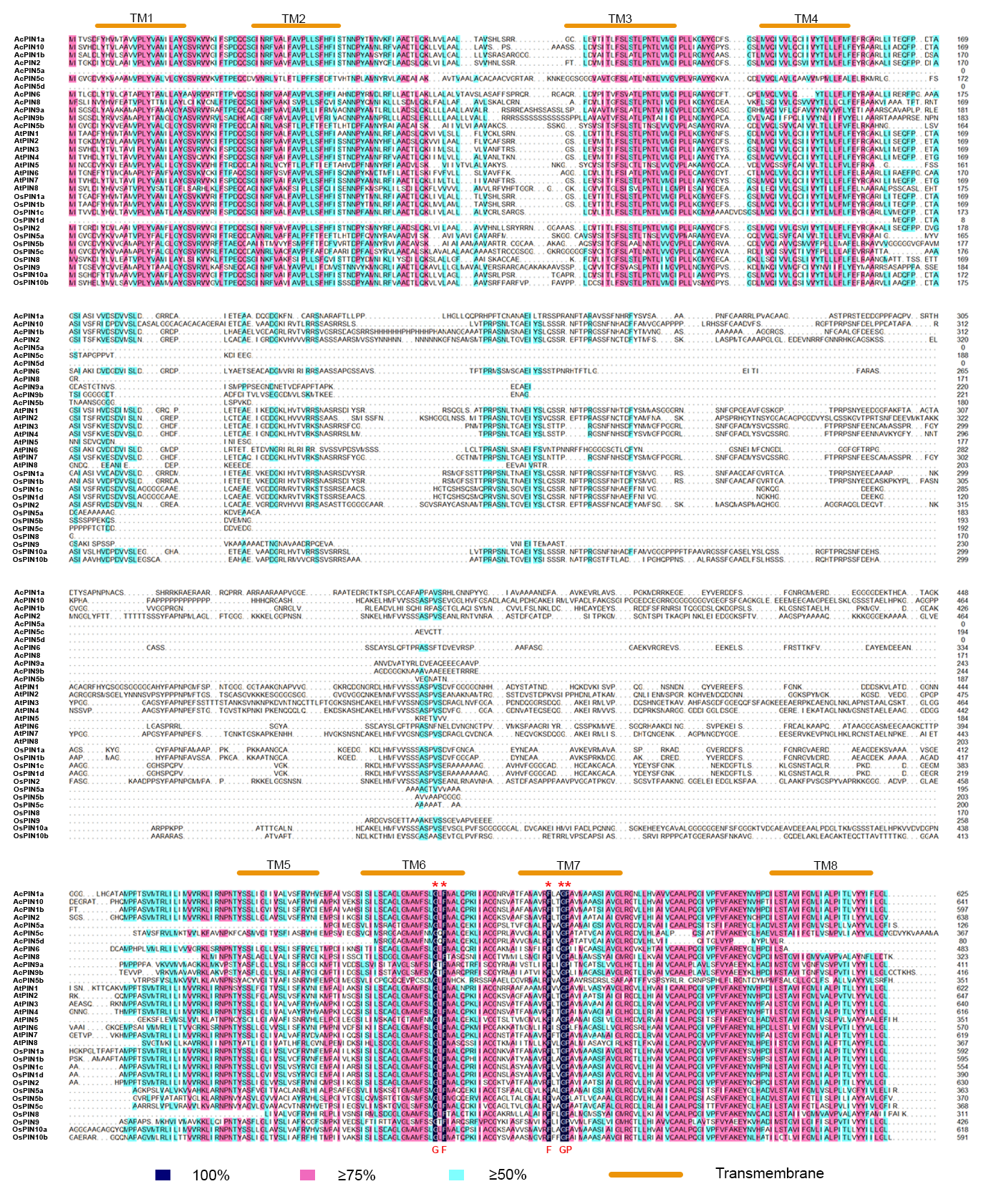

Supplement: Supplemental Information 6 — Identical, conservative and block of similar amino acid residues are shaded in deep blue, pink and light green, respectively. The transmembrane (TM) regions are marked by orange ellipses. Asterisks () indicate the completely conserved amino acid. [file peerj-09-11410-s006.png]

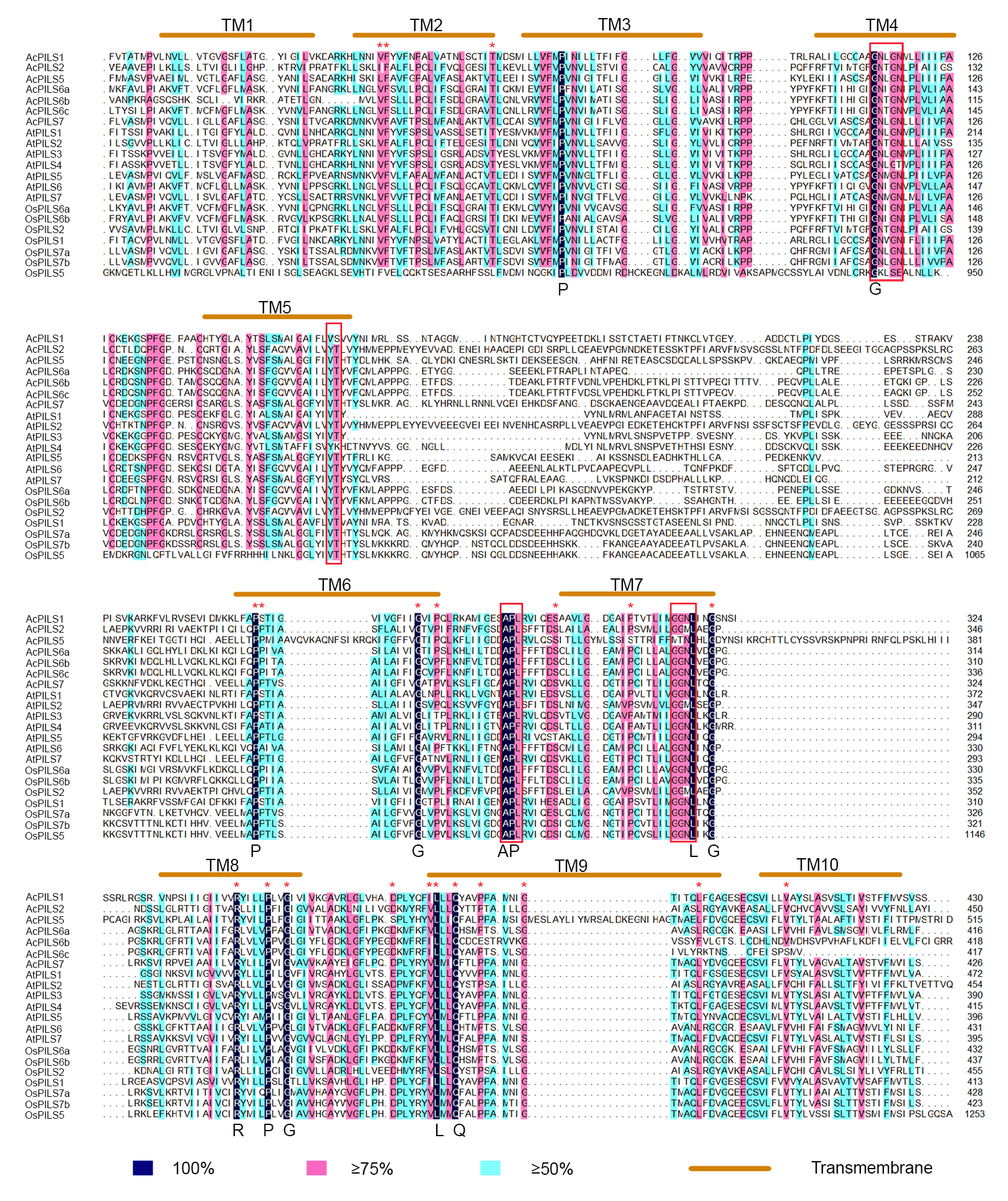

Supplement: Supplemental Information 7 — Identical, conservative and block of similar amino acid residues are shaded in deep blue, pink and light green, respectively. The red rectangles indicate the conserved domain at N-terminus or C-terminus. The transmembrane (TM) regions are marked by orange ellipses. [file peerj-09-11410-s007.png]

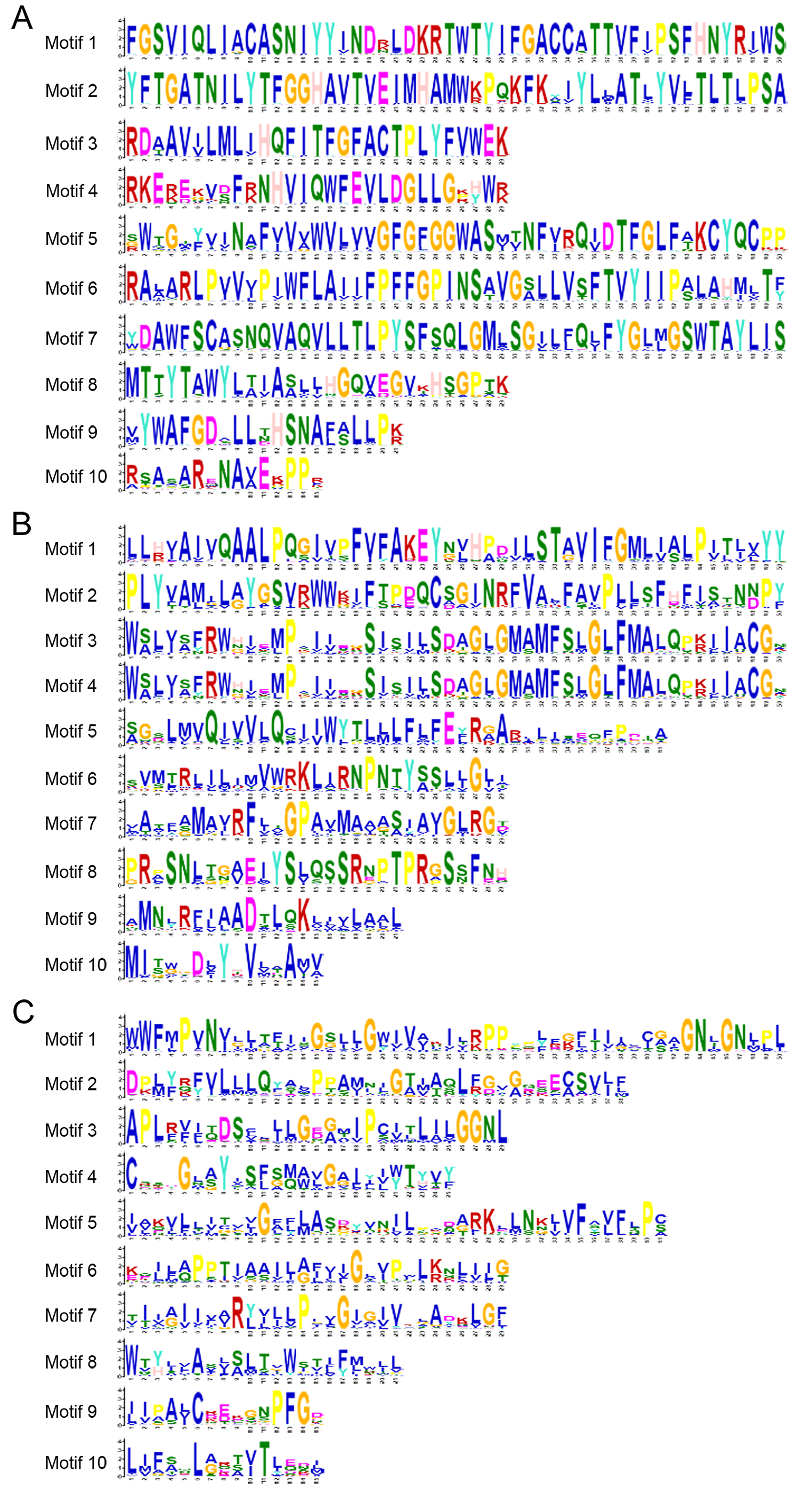

Supplement: Supplemental Information 8 — The motif sequences of AUX (A), PIN (B) and PILS(C) proteins in Fig. 6 are shown. Conversed protein motifs were identified by MEME motif search tool. The height of each character in the motif sequences represent the conservation of amino acid identity. [file peerj-09-11410-s008.png]

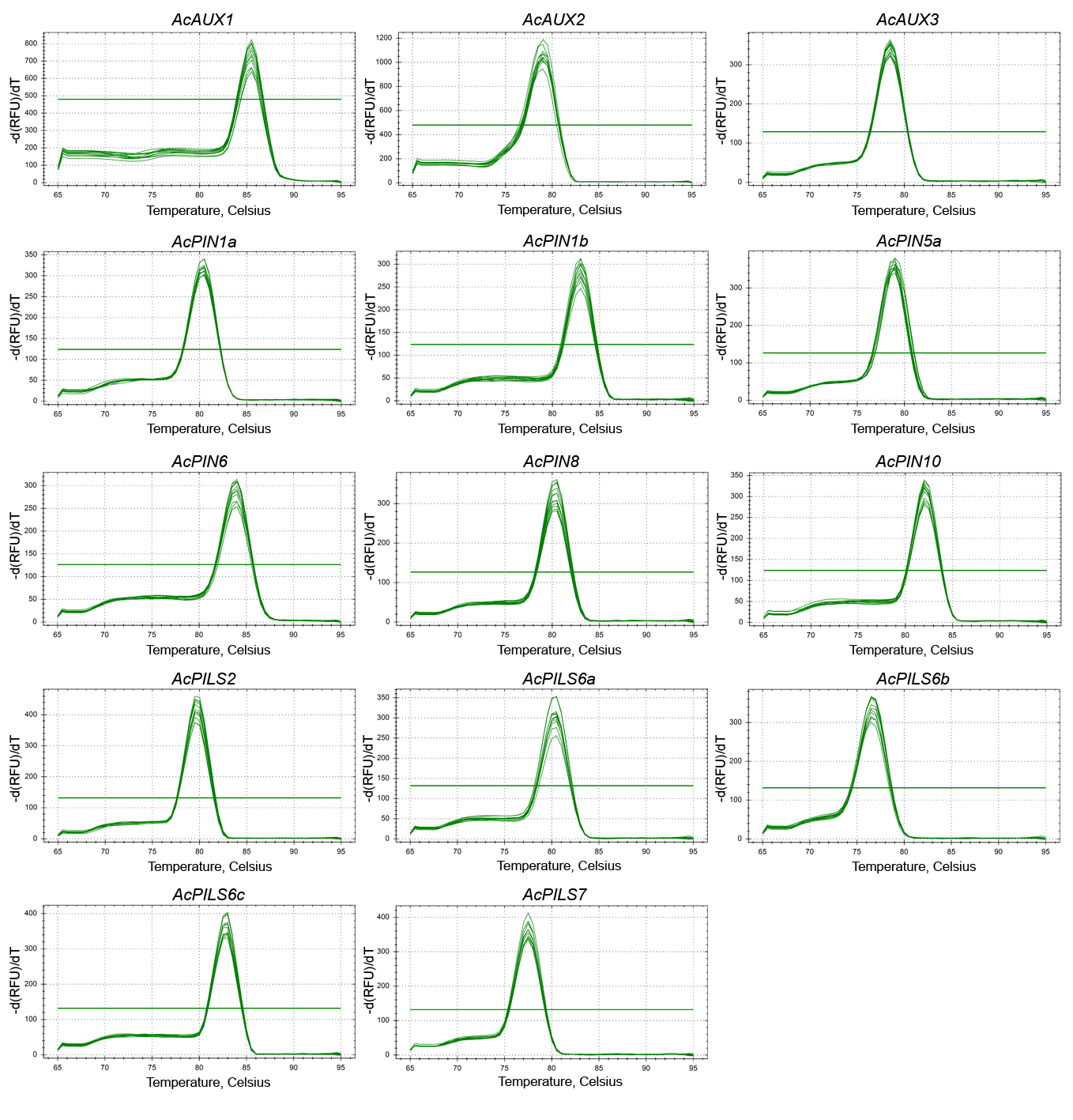

Supplement: Supplemental Information 9 [file peerj-09-11410-s009.png]
