# Supplementary material for: Characterization of auxin transporter AUX, PIN and PILS gene families in pineapple and evaluation of expression profiles during reproductive development and under abiotic stresses
Source: PeerJ. 2021 Jun 22;9:e11410. doi: 10.7717/peerj.11410 (PMC8231336; doi:10.7717/peerj.11410)
Supplement: Supplemental Information 10 [file peerj-09-11410-s010.doc]

**Table S1 Expression data of *AcAUX*, *AcPIN* and *AcPILS* genes in several tissues and fruits.**

| **Genes** | **R** | **L** | **F** | **S1** | **S2** | **S3** | **S4** | **S5** | **S6** |
| --- | --- | --- | --- | --- | --- | --- | --- | --- | --- |
| ***AcAUX1*** | 1.10 | 0.33 | 0.26 | -2.01 | -2.04 | -1.66 | -1.38 | -1.56 | -3.55 |
| ***AcAUX2*** | -1.18 | 0 | 0 | -6.80 | -5.90 | 0 | 0 | 0 | 0 |
| ***AcAUX3*** | 2.15 | -1.26 | -1.22 | -2.29 | -2.38 | -3.29 | -2.92 | -4.00 | -6.67 |
| ***AcPIN1a*** | 0.309 | -0.52 | -0.59 | -2.46 | -2.00 | -2.52 | -3.17 | -4.23 | -3.32 |
| ***AcPIN1b*** | -1.29 | -4.31 | -4.10 | -2.86 | -2.67 | -3.63 | -4.65 | -4.90 | -7.62 |
| ***AcPIN2*** | -0.08 | 0 | -9.87 | 0 | 0 | 0 | 0 | 0 | 0 |
| ***AcPIN5a*** | 0 | -5.66 | -6.54 | -1.15 | -0.61 | -2.79 | -5.05 | 0 | 0 |
| ***AcPIN5b*** | 0 | -6.13 | -6.69 | -2.02 | -1.77 | -3.55 | -6.52 | -6.72 | 0 |
| ***AcPIN5c*** | -3.20 | 0 | -9.70 | 0 | 0 | 0 | 0 | 0 | 0 |
| ***AcPIN5d*** | -5.52 | 0 | 0 | 0 | 0 | 0 | 0 | 0 | 0 |
| ***AcPIN6*** | 0 | -5.78 | -4.76 | 0 | -6.14 | -6.96 | -7.3 | 0 | 0 |
| ***AcPIN8*** | -1.74 | -2.93 | -2.07 | -4.45 | -4.05 | -5.06 | -4.02 | -3.33 | -6.00 |
| ***AcPIN9a*** | -4.87 | 0 | 0 | 0 | 0 | 0 | 0 | 0 | 0 |
| ***AcPIN9b*** | -7.30 | -1.41 | -1.28 | -5.30 | 0 | 0 | 0 | 0 | -9.69 |
| ***AcPIN10*** | -2.05 | 0.96 | 0.89 | -0.85 | -0.88 | -1.19 | -1.06 | -1.65 | -2.64 |
| ***AcPILS1*** | -3.05 | -1.77 | -1.92 | -1.03 | -2.78 | -3.99 | -3.16 | -5.60 | -2.36 |
| ***AcPILS2*** | -0.33 | -0.36 | -0.35 | 1.37 | 1.87 | 2.28 | 3.05 | 3.60 | 4.10 |
| ***AcPILS5*** | -1.11 | -0.67 | -0.27 | 0.11 | -0.61 | -0.94 | -1.73 | -2.25 | -3.69 |
| ***AcPILS6a*** | 0.34 | 1.94 | 1.98 | 0.67 | 1.03 | 0.95 | 2.13 | 0.99 | 0.04 |
| ***AcPILS6b*** | -0.85 | 1.75 | 1.69 | 3.82 | 4.19 | 4.31 | 4.45 | 4.78 | 3.83 |
| ***AcPILS6c*** | -1.40 | 0.65 | 0.66 | 1.75 | 2.08 | 1.78 | 1.92 | 2.60 | 2.35 |
| ***AcPILS7*** | 0.58 | 3.12 | 3.23 | 1.84 | 1.94 | 0.39 | -0.11 | -1.38 | -1.08 |

The numbers on the tables indicate the average log signal values for these 22 genes in the tissues examined. **R**, roots; **L**, leaves; **F**, flower; **S1-S6**, different stages of fruit development.
