# Supplementary material for: Characterization of auxin transporter AUX, PIN and PILS gene families in pineapple and evaluation of expression profiles during reproductive development and under abiotic stresses
Source: PeerJ. 2021 Jun 22;9:e11410. doi: 10.7717/peerj.11410 (PMC8231336; doi:10.7717/peerj.11410)
Supplement: Supplemental Information 11 [file peerj-09-11410-s011.doc]

**Table S2 Expression data of *AcAUX*, *AcPIN* and *AcPILS* genes in various flower organs.**

| **Genes** | **Ca1** | **Ca2** | **Ca3** | **Ca4** | **Pe1** | **Pe2** | **Pe3** | **St1** | **St2** | **St3** | **St4** | **St5** | **Ov1** | **Ov2** | **Ov3** | **Ov4** | **Ov5** | **Ov6** | **Ov7** |
| --- | --- | --- | --- | --- | --- | --- | --- | --- | --- | --- | --- | --- | --- | --- | --- | --- | --- | --- | --- |
| ***AcAUX1*** | 0.57 | 0.54 | 0.83 | 0.97 | -1.10 | 0.57 | 1.09 | -1.07 | -0.61 | 0 | 0.62 | -0.28 | 0.37 | 0.14 | -0.09 | -0.13 | -0.26 | 0.31 | 0.43 |
| ***AcAUX2*** | -4.89 | -8.05 | 0 | -7.39 | -1.60 | -4.63 | 0 | 0.55 | -0.2 | -0.98 | -1.56 | -4.66 | 2.40 | 2.15 | 2.06 | 1.59 | 1.40 | 0.87 | 0.61 |
| ***AcAUX3*** | -3.31 | -1.88 | -1.13 | -1.45 | -5.42 | -1.50 | 1.47 | -5.44 | -5.51 | -5.04 | -8.09 | -4.13 | -3.65 | -2.47 | -1.50 | -2.63 | -2.63 | -3.53 | -2.21 |
| ***AcPIN1a*** | -3.36 | -2.37 | -2.98 | -3.64 | -2.43 | 0.39 | 1.56 | -2.2 | -2.4 | -2.67 | -3.57 | -1.29 | 0.54 | 0.31 | -0.42 | 0.08 | -0.44 | -0.32 | -1.33 |
| ***AcPIN1b*** | -0.70 | -2.1 | -1.79 | -1.84 | 0.39 | -1.34 | -3.29 | 1.63 | 0.76 | -0.61 | -1.21 | -2.96 | 1.73 | 1.68 | 1.43 | 1.16 | 0.99 | 0.09 | 0.22 |
| ***AcPIN2*** | 0.58 | -0.51 | -0.77 | -0.4 | 0 | 0 | 0 | 0 | -8.17 | 0 | -7.45 | 0 | -9.95 | 0 | 0 | 0 | 0 | 0 | 0 |
| ***AcPIN5a*** | 0 | -4.13 | -6.33 | -5.36 | 0 | -5.1 | 0 | 0 | 0 | 0 | 0 | -8.29 | 0 | -9.1 | 0 | 0 | 0 | 0 | 0 |
| ***AcPIN5b*** | 0 | -3.51 | -5.93 | -4.51 | 0 | -4.68 | -7.3 | 0 | 0 | 0 | -8.99 | 0 | 0 | 0 | 0 | 0 | 0 | 0 | 0 |
| ***AcPIN5c*** | 0 | 0 | 0 | 0 | 0 | 0 | -8.25 | 0 | -9.75 | 0 | -8.88 | -9.30 | 0 | 0 | 0 | 0 | 0 | 0 | 0 |
| ***AcPIN5d*** | 0 | 0 | 0 | 0 | 0 | 0 | 0 | 0 | 0 | 0 | 0 | 0 | 0 | 0 | 0 | 0 | 0 | 0 | 0 |
| ***AcPIN6*** | -2.57 | -2.45 | -6.02 | -5.72 | -0.59 | 0.23 | -6.36 | -3.94 | -3.92 | -4.99 | -4.76 | -5.88 | -0.04 | -0.61 | -1.59 | -1.54 | -2.22 | -3.18 | -5.05 |
| ***AcPIN8*** | -4.37 | -3.48 | -3.55 | -4.52 | -3.13 | -1.93 | -3.47 | -3.58 | -3.48 | -3.71 | -3.46 | -3.42 | -1.46 | -1.35 | -1.97 | -1.89 | -2.24 | -2.60 | -2.21 |
| ***AcPIN9a*** | 0 | 0 | 0 | 0 | 0 | 0 | 0 | -8.69 | 0 | -7.11 | -5.40 | 0 | 0 | 0 | 0 | 0 | 0 | 0 | 0 |
| ***AcPIN9b*** | 0 | -6.72 | -5.81 | -5.49 | -6 | -6.75 | -4.22 | -0.36 | -0.14 | 1.00 | 0.96 | 1.73 | -7.39 | -8.83 | 0 | 0 | -6.55 | -5.55 | -5.56 |
| ***AcPIN10*** | 0.96 | 1.43 | 1.17 | 1.20 | 1.18 | 2.03 | 2.41 | -3.49 | -2.65 | -2.69 | -0.91 | 0.47 | -1.24 | -0.51 | -0.63 | -1.85 | -1.40 | -1.01 | -0.72 |
| ***AcPILS1*** | -0.28 | -0.38 | -0.22 | -0.9 | -1.65 | -3.40 | -0.07 | -0.19 | -1 | 2.63 | 3.76 | 0.62 | -2.18 | -1.27 | -1.3 | -1.04 | -0.76 | -0.53 | -1.43 |
| ***AcPILS2*** | 2.24 | 2.94 | 2.52 | 2.51 | 1.54 | 2.19 | 3.04 | 1 | 0.76 | 0.87 | 1.25 | 1.56 | 0.69 | 0.88 | 0.80 | 0.72 | 0.62 | 0.81 | 1.35 |
| ***AcPILS5*** | -1.25 | -1.37 | -1.01 | -1.77 | -1.04 | -0.75 | 0.43 | -2.22 | -2.42 | -2.40 | -2.12 | -2.65 | -2.70 | -2.23 | -3.05 | -1.78 | -1.58 | -2.17 | -1.96 |
| ***AcPILS6a*** | 1.16 | 0.93 | 1.29 | 0.89 | 0.14 | 0.79 | 3.80 | -0.05 | -0.05 | 0.48 | 0.16 | 2.65 | -0.59 | -0.68 | -0.87 | -0.70 | -0.97 | -0.96 | -1.10 |
| ***AcPILS6b*** | 1.04 | 2.375 | 1.96 | 1.77 | 1.64 | 2.16 | 3.96 | 0.82 | 0.88 | 0.76 | 1.81 | 2.61 | 0.032 | -0.03 | -0.65 | -0.48 | -0.59 | 0.59 | -0.33 |
| ***AcPILS6c*** | -0.72 | 0.86 | 0.50 | 0.29 | 0.17 | 0.39 | 2.36 | -0.69 | -0.49 | -0.75 | 0.267 | 1.15 | -1.51 | -1.44 | -2.19 | -2.09 | -1.71 | -0.85 | -1.27 |
| ***AcPILS7*** | 1.87 | 2.61 | 2.40 | 1.81 | 1.18 | 3.44 | 4.202 | -5.62 | -3.65 | -2.65 | -0.74 | -2.5 | -0.70 | 0.15 | 0.22 | -0.48 | 0.21 | 0.45 | 0.47 |

The numbers on the tables indicate the average log signal values for these 22 genes in the flower organs**. Ca**, calyx; **Pe**, petal; **St**, stamen; **Ov**, ovule. The numbers following the tissues indicated different developmental stages.
