# Supplementary material for: Characterization of auxin transporter AUX, PIN and PILS gene families in pineapple and evaluation of expression profiles during reproductive development and under abiotic stresses
Source: PeerJ. 2021 Jun 22;9:e11410. doi: 10.7717/peerj.11410 (PMC8231336; doi:10.7717/peerj.11410)
Supplement: Supplemental Information 12 [file peerj-09-11410-s012.doc]

**Table S3 Data for expression comparison of** ***AUX*(A), *PIN*(B) and *PILS*(C) genes between pineapple, Arabido*psis* and rice in different organs**

**(A)**

| **Genes** | **Root** | **Leaf** | **Flower** | **Seeds/Fruits** |
| --- | --- | --- | --- | --- |
| ***AtAUX1*** | 2.45 | 1.53 | 2.45 | 0.90 |
| ***AtLAX1*** | -0.06 | 0.646 | 0.056 | -0.47 |
| ***AcAUX1*** | 1.10 | 0.33 | 0.26 | -1.38 |
| ***OsAUX1*** | 2.77 | 1.13 | 3.34 | 1.44 |
| ***OsAUX2*** | 1.27 | -0.28 | 1.00 | 0.73 |
| ***AtLAX2*** | 0.42 | -0.34 | 1.03 | 0.44 |
| ***AcAUX2*** | -1.18 | 0 | 0 | 0 |
| ***OsAUX3*** | -1.18 | -3.64 | 0.33 | -1.39 |
| ***OsAUX4*** | 1.18 | -3.26 | 0.48 | -1.60 |
| ***OsAUX5*** | -0.31 | -1.49 | 0.15 | -1.20 |
| ***AtLAX3*** | 2.36 | -1.88 | -0.11 | -0.71 |
| ***AcAUX3*** | 2.15 | -1.26 | -1.22 | -2.29 |

**(B)**

| **Genes** | **Root** | **Leaf** | **Flower** | **Seeds/Fruits** |
| --- | --- | --- | --- | --- |
| ***AtPIN1*** | -0.01 | -1.48 | 0.44 | -0.26 |
| ***OsPIN1a*** | -0.66 | -3.99 | -1.65 | -2.47 |
| ***OsPIN1b*** | 1.46 | -1.60 | 1.09 | -0.76 |
| ***OsPIN1c*** | -1.61 | -4.04 | 0.16 | -2.70 |
| ***AcPIN1a*** | 0.31 | -0.52 | -0.59 | -2.00 |
| ***AcPIN10*** | -2.05 | 0.95 | 0.89 | -0.85 |
| ***AcPIN1b*** | -1.29 | -4.31 | -4.10 | -2.67 |
| ***AcPIN2*** | -0.08 | 0 | -9.87 | 0 |
| ***AtPIN2*** | 1.00 | -3.46 | -2.32 | -1.76 |
| ***OsPIN2*** | 0.67 | -3.61 | -3.93 | -2.98 |
| ***AtPIN3*** | 0.15 | 0.83 | 1.36 | 0.53 |
| ***AtPIN4*** | -0.55 | 1.25 | 0.60 | -0.90 |
| ***AtPIN6*** | -2.14 | -1.74 | -0.64 | -1.51 |
| ***AcPIN6*** | 0 | -5.78 | -4.76 | 0 |
| ***AtPIN7*** | -1.08 | -0.54 | 0.384 | -1.00 |
| ***AtPIN8*** | -3.78 | -3.11 | -2.29 | -2.10 |
| ***OsPIN8*** | -4.24 | -4.28 | -1.91 | -3.60 |
| ***AcPIN8*** | -1.74 | -2.92 | -2.07 | -3.33 |
| ***OsPIN9*** | -1.23 | -3.44 | -1.54 | -3.63 |
| ***AcPIN9a*** | -4.87 | 0 | 0 | 0 |
| ***AcPIN9b*** | -7.30 | -1.41 | -1.28 | 0 |
| ***AtPIN5*** | -4.13 | -3.30 | -3.07 | -2.14 |
| ***OsPIN5a*** | -3.53 | -0.22 | -1.47 | -1.45 |
| ***OsPIN5b*** | -4.55 | -4.28 | -3.03 | -3.48 |
| ***AcPIN5a*** | 0 | -5.66 | -6.54 | 0 |
| ***AcPIN5c*** | -3.20 | 0 | -9.70 | 0 |
| ***AcPIN5d*** | -5.52 | 0 | 0 | 0 |
| ***OsPIN10a*** | -3.64 | -3.03 | 2.30 | -0.29 |
| ***OsPIN10b*** | -3.97 | -4.08 | -3.52 | -3.46 |
| ***AcPIN5b*** | 0 | -6.13 | -6.68 | 0 |

**(C)**

| **Genes** | **Root** | **Leaf** | **Flower** | **Seeds/Fruits** |
| --- | --- | --- | --- | --- |
| ***AcPILS2*** | -0.33 | -0.36 | -0.34 | 4.10 |
| ***AtPILS2*** | 0.71 | 0.53 | 0.45 | 1.56 |
| ***OsPILS2*** | 3.05 | 3.89 | 3.21 | 3.17 |
| ***AcPILS1*** | -3.05 | -1.77 | -1.92 | -1.03 |
| ***AtPILS3*** | -1.06 | 0.62 | 1.39 | 1.14 |
| ***AtPILS4*** | -4.08 | -3.83 | -2.30 | -2.65 |
| ***AtPILS5*** | 1.63 | -1.12 | 2.25 | 0.89 |
| ***AcPILS5*** | -1.10 | -0.67 | -0.27 | 0.11 |
| ***OsPILS5*** | -1.15 | -0.86 | -0.70 | -1.45 |
| ***OsPILS7a*** | -0.26 | -1.61 | -1.32 | -0.92 |
| ***AcPILS7*** | 0.58 | 3.12 | 3.23 | 1.94 |
| ***AtPILS6*** | -0.13 | -1.22 | -0.68 | 0.98 |
| ***AcPILS6b*** | -0.85 | 1.75 | 1.69 | 4.78 |
| ***AcPILS6c*** | -1.40 | 0.65 | 0.66 | 2.60 |
| ***AcPILS6a*** | 0.34 | 1.94 | 1.98 | 2.13 |
| ***OsPILS6a*** | 1.53 | 2.20 | 0.41 | 0.49 |
| ***OsPILS6b*** | -3.93 | -4.24 | -3.72 | -2.04 |

The numbers on the tables indicate the average log values of these 22 genes. Red, green, yellow and light yellow boxes indicate high (more than 1), moderate (between 0 and 1), low (between -1 and 0), and extremely low (less than -1 or no signature is found) expression levels, respectively.
