# Supplementary material for: Characterization of auxin transporter AUX, PIN and PILS gene families in pineapple and evaluation of expression profiles during reproductive development and under abiotic stresses
Source: PeerJ. 2021 Jun 22;9:e11410. doi: 10.7717/peerj.11410 (PMC8231336; doi:10.7717/peerj.11410)
Supplement: Supplemental Information 13 [file peerj-09-11410-s013.doc]

**Table S4 Total reads and sequencing depth for 28 samples.**

| **Samples** | **Total reads (G)** | **Sequencing Depth** |
| --- | --- | --- |
| R | 6.62 | 12.58555133 |
| F | 7.42 | 14.10646388 |
| L | 4.15 | 7.88973384 |
| S1 | 2.57 | 4.885931559 |
| S2 | 5.57 | 10.58935361 |
| S3 | 5.58 | 10.60836502 |
| S4 | 6.85 | 13.02281369 |
| S5 | 4.97 | 9.448669202 |
| S6 | 10.69 | 20.32319392 |
| Ca1 | 4.7 | 8.935361217 |
| Ca2 | 4.5 | 8.55513308 |
| Ca3 | 5.2 | 9.885931559 |
| Ca4 | 5.3 | 10.07604563 |
| Pe1 | 4.6 | 8.745247148 |
| Pe2 | 4 | 7.604562738 |
| Pe3 | 4.3 | 8.174904943 |
| St1 | 3.4 | 6.463878327 |
| St2 | 4.6 | 8.745247148 |
| St3 | 3.9 | 7.414448669 |
| St4 | 4 | 7.604562738 |
| St5 | 4.8 | 9.125475285 |
| Ov1 | 5 | 9.505703422 |
| Ov2 | 5 | 9.505703422 |
| Ov3 | 5 | 9.505703422 |
| Ov4 | 4.9 | 9.315589354 |
| Ov5 | 5.2 | 9.885931559 |
| Ov6 | 5.1 | 9.69581749 |
| Ov7 | 5.3 | 10.07604563 |

**R**, roots; **L**, leaves; **F**, flowers; **S1-S6**, different stages of fruit development.

**Ca**, calyx; **Pe**, petal; **St**, stamen; **Ov**, ovule. The numbers following the tissues indicated different developmental stages.
