# Supplementary material for: Characterization of auxin transporter AUX, PIN and PILS gene families in pineapple and evaluation of expression profiles during reproductive development and under abiotic stresses
Source: PeerJ. 2021 Jun 22;9:e11410. doi: 10.7717/peerj.11410 (PMC8231336; doi:10.7717/peerj.11410)
Supplement: Supplemental Information 14 [file peerj-09-11410-s014.docx]

**Table S5 The gene-specific primers for qRT-PCR in this study.**

| **Primers** | **Sequences ( 5' to 3' )** |
| --- | --- |
| *AcAUX1*-F | CAGCTGTTCTACGGCTTCAT |
| *AcAUX1*-R | CACTGGATGACGTGGTTCTT |
| *AcAUX2*-F | CCGTCTCCTTCGCACTTT |
| *AcAUX2*-R | CCTACCACTTTCTCTCTCTCTTTC |
| *AcAUX3*-F | GAGATGGAAAGGGAAGGAGAAG |
| *AcAUX3*-R | ATGAGTATGGCAGTGTGAGAAG |
| *AcPIN1a*-F | CTCCCGACATCTTGGAAACA |
| *AcPIN1a*-R | CTCCTTCCGTCGATCCATTT |
| *AcPIN10*-F | TCCGATCACACTGGTCTACT |
| *AcPIN10*-R | GTCTCAATCTGGATGGCTACTT |
| *AcPIN1b*-F | CCAGTGTAATGACTCGGCTTAT |
| *AcPIN1b*-R | AAGGAGATCAGAGACCAGATGA |
| *AcPIN5a*-F | GAATCACTTGGGCCTTCATTTC |
| *AcPIN5a*-R | CTGCCCTGGACATAATTAGCA |
| *AcPIN6*-F | CTAGTGGTGGTGCTGAGAAAG |
| *AcPIN6*-R | CGTCCATCTCATACGCATCTAC |
| *AcPIN8*-F | CTGGCATACAACTTCCTCCTT |
| *AcPIN8*-R | TCCTTCGCCATAGTTCCTTATTT |
| *AcPILS2*-F | CCGTGATCATGACCGGATTT |
| *AcPILS2*-R | CATTGAGCGAAGGAGAGGTAAG |
| *AcPILS6a*-F | GAGAATCCATCCTCGCATCAA |
| *AcPILS6a*-R | AACGTACTTGGAGGCCATTAG |
| *AcPILS6b*-F | CGAAACTACCAACGAGGACTT |
| *AcPILS6b*-R | GGGCACAGTAGTGGTGATTT |
| *AcPILS6c*-F | CTCCAGTAGGGCTTGGAATTTA |
| *AcPILS6c*-R | GAGGAGGAGGATGAATCTGAAC |
| *AcPILS7*-F | CTCGGCAATCTACTGCTGATAA |
| *AcPILS7*-R | CATCGAGAAAGACGCGTAAGA |

F, Forward primer; R, Reverse primer
